# Metabolomics for Diagnosis and Prognosis of Uterine Diseases? A Systematic Review

**DOI:** 10.3390/jpm10040294

**Published:** 2020-12-21

**Authors:** Janina Tokarz, Jerzy Adamski, Tea Lanišnik Rižner

**Affiliations:** 1 Research Unit Molecular Endocrinology and Metabolism, Helmholtz Zentrum München, German Research Centre for Environmental Health, Ingolstädter Landstr. 1, 85764 Neuherberg, Germany; janina.tokarz@helmholtz-muenchen.de (J.T.); adamski@helmholtz-muenchen.de (J.A.); 2 German Centre for Diabetes Research, Ingolstaedter Landstrasse 1, 85764 Neuherberg, Germany; 3 Lehrstuhl für Experimentelle Genetik, Technische Universität München, Freising-Weihenstephan, 85764 Neuherberg, Germany; 4 Department of Biochemistry, Yong Loo Lin School of Medicine, National University of Singapore, Singapore 117596, Singapore; 5 Institute of Biochemistry, Faculty of Medicine, University of Ljubljana, Vrazov trg 2, 1000 Ljubljana, Slovenia

**Keywords:** adenomyosis, algorithms, biomarker, biomarker discovery, cervical cancer, endometriosis, endometrial cancer, leiomyoma, omics, uterine fibroids

## Abstract

This systematic review analyses the contribution of metabolomics to the identification of diagnostic and prognostic biomarkers for uterine diseases. These diseases are diagnosed invasively, which entails delayed treatment and a worse clinical outcome. New options for diagnosis and prognosis are needed. PubMed, OVID, and Scopus were searched for research papers on metabolomics in physiological fluids and tissues from patients with uterine diseases. The search identified 484 records. Based on inclusion and exclusion criteria, 44 studies were included into the review. Relevant data were extracted following the PRISMA (Preferred Reporting Items for Systematic Reviews and Meta-Analysis) checklist and quality was assessed using the QUADOMICS tool. The selected metabolomics studies analysed plasma, serum, urine, peritoneal, endometrial, and cervico-vaginal fluid, ectopic/eutopic endometrium, and cervical tissue. In endometriosis, diagnostic models discriminated patients from healthy and infertile controls. In cervical cancer, diagnostic algorithms discriminated patients from controls, patients with good/bad prognosis, and with/without response to chemotherapy. In endometrial cancer, several models stratified patients from controls and recurrent from non-recurrent patients. Metabolomics is valuable for constructing diagnostic models. However, the majority of studies were in the discovery phase and require additional research to select reliable biomarkers for validation and translation into clinical practice. This review identifies bottlenecks that currently prevent the translation of these findings into clinical practice.

## 1. Introduction

### 1.1. Rationale for the Review

Metabolomics is rapidly gaining importance in medicine [1]. It has great potential for the development of diagnostic and prognostic approaches, to be applied to monitor disease progression, and to determine the effects of therapeutic agents. Moreover, metabolomic profiling can also contribute to a detailed understanding of pathological molecular mechanisms, which might allow identification of new drug targets [2]. In this manner, metabolomics can tackle the unmet need for better diagnosis and personalized treatment of uterine diseases.

In the field of gynecology and reproduction, the first metabolomics study with the aim to identify novel biomarkers of endometriosis was published in 2012 [3]. Since then, there have been a number of metabolomics studies dealing with uterine diseases, with 50 published in 2018, 45 in 2019, and 16 to July 2020, based on the PubMed search (Table 1). 

### 1.2. Metabolomics

Metabolomics is the most recent addition to the group of ‘-omics’ technologies, which include genomics (sequencing of all genes), transcriptomics (determining all transcripts), and proteomics (measuring all proteins) [4]. Ideally, metabolomics strives to measure all metabolites present in a given biological sample. Metabolites are small molecules with molecular mass below 1200 Da, such as sugars, amino acids, acylcarnitines, organic acids, and lipids. All of the metabolites together in a cell, tissue, or organism are designated the ‘metabolome’ [5]. 

As metabolites are intermediates as well as final downstream products of cellular processes, metabolomics is closest to the actual phenotype compared to the other -omics techniques. With the advent of advanced analytical methods with high sensitivities and the resultant wide metabolite coverage, metabolomics is increasingly applied in human health and biomedical research [6]. Metabolomics has been carried out for a large variety of different sample types, including human body fluids, tissues, and feces [7].

#### 1.2.1. Nontargeted and Targeted Metabolomics

**Figure 1:**
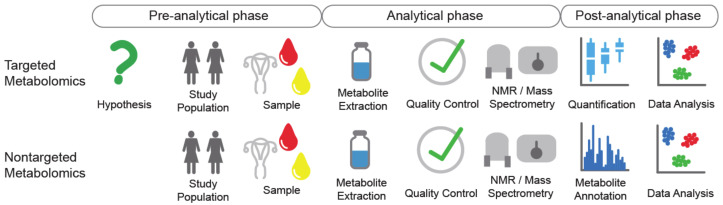
Flow diagram of metabolomics approaches. Targeted and nontargeted metabolomics are both divided into pre-analytical, analytical, and post-analytical phases.

For metabolomics analysis, two approaches with different objectives are used [8]: nontargeted and targeted metabolomics. Nontargeted metabolomics is also known as profiling metabolomics, as this is a hypothesis-free approach that aims to simultaneously detect as many metabolites as possible in a given sample. Depending on the analytical platform, nontargeted metabolomics measures metabolites from a wide range of metabolite classes [6], which are annotated after the measurements (Figure 1). Thus, the detection of unknown metabolites that have not been annotated yet in metabolite databases is a common phenomenon in nontargeted metabolomics. A downside of nontargeted metabolomics is that this technique does not allow absolute quantification, but can provide at best only semiquantitative data [6].

Targeted metabolomics, in contrast, aims to quantify the absolute concentrations of a predefined set of metabolites. Therefore, targeted metabolomics is a hypothesis-driven approach [6] (Figure 1). As all of the measured metabolites are pre-selected, a standard calibration curve for accurate quantification can be prepared for each metabolite of interest. Internal standards labeled with stable isotopes are added at a constant amount to all of the samples, to compensate for any analytical interference. Targeted metabolomics methods can be validated in terms of precision, accuracy, and calibration ranges [9]. With its advantages including validated analytical performance, and with the data delivered as absolute concentrations, targeted metabolomics is often used for biomarker validation [6]. The drawback of targeted metabolomics is the limited set of simultaneously quantified metabolites, which increases the risk of missing interesting biological processes. 

Although nontargeted and targeted metabolomics require different sample processing protocols, the combined application of both of these analytical techniques to aliquots of the same sample is advantageous. Unfortunately, none of the current analytical methods can cover the entire metabolome on its own [10]. Thus, the integration of different metabolomics techniques (i.e.; multiplatform approaches) is necessary to achieve high metabolite coverage combined with quantitative data [10,11]. Most frequently, for metabolomics measurements, mass spectrometry (MS) or nuclear magnetic resonance (NMR) spectroscopy are used, with or without being coupled to metabolite separation techniques [7]. 

#### 1.2.2. Mass Spectrometry

Mass spectrometry (MS) is an analytical technique that measures the mass-to-charge ratios (*m/z*) of ionized molecules. The molecules are ionized in the ion source of the mass spectrometer, and then transferred to the core of the instrument, where the ions are separated based on their mass-to-charge ratios. The ions are detected by charge detection devices, such as electron multiplying detectors, and the data are recorded as spectra of the ion intensity as a function of the mass-to-charge ratio [12].

Modern MS instruments offer high mass accuracy, high resolution, high dynamic range, high sensitivity, and fast scan rates [7]. MS instruments can be successfully operated for metabolomics analysis in flow injection analysis mode, i.e.; the sample is directly injected into the apparatus [13]. However, to increase the resolution of metabolites, the mass spectrometer is usually coupled to a separation technique, such as gas chromatography (GC), liquid chromatography (LC), or ultra-high performance liquid chromatography (UPLC) [6,14]. The chromatographic separation segregates the individual metabolites and reduces the levels of perturbing substances, which leads to an increase in the relative intensity of the analyte signal [7]. 

Despite these advantages of MS, this technique also has two important disadvantages. First, MS is a destructive technology, i.e.; the sample is not available anymore after the analysis. Second, high-throughput metabolomics analysis can impair the quality of the measurements due to accumulation of impurities inside the mass spectrometer [15].

#### 1.2.3. Nuclear Magnetic Resonance Spectroscopy

Nuclear magnetic resonance spectroscopy (NMR) is a technique used to measure the magnetic fields around atoms. Any molecule that contains one or more atoms with nonzero magnetic moments can be detected, such as ^1^H, ^13^C, ^14^N, and ^31^P, although ^1^H NMR is most frequently used for metabolomics [16]. The sample is inserted into a powerful magnetic field, the molecules are excited by radio waves, and the signals are detected by sensitive radio receivers. Each signal corresponds to a specific atom in a molecule. Data is usually recorded as a spectrum of intensity versus frequency [17].

Compared to MS, NMR is less sensitive, but is superior in terms of the structural information content, robustness, and reproducibility [18,19]. NMR is nondestructive, and the full and correct functioning of the instrument is therefore not impaired by sample components [7]. By definition, NMR is a quantitative spectroscopic tool that allows for quantification of metabolites without preparation of analyte standard calibration curves [19,20]. Additionally, sample preparation for NMR-based metabolomics is simpler and faster for some matrices, such as urine or plasma, than for MS-based metabolomics [10,18]. NMR can detect lipids with high resolution and other metabolites with medium to high abundance (e.g.; amino acids, organic acids, sugars) [10,19]. However, NMR analysis provides information only on known metabolites, i.e.; metabolites that are annotated in databases [7]. 

### 1.3. Uterine Diseases

Uterine diseases include benign pathologies of myometrium, the very frequent uterine fibroids (myoma uteri, uterus myomatosus), adenomyosis, endometriosis, and malignant diseases, such as cervical and endometrial cancers. These diseases are very common and can jeopardize the reproductive status of women. During their reproductive age, up to 70% of women develop uterine fibroids, up to 20% are afflicted with adenomyosis, up to 10% suffer from endometriosis, and 35% to 50% show infertility associated with endometriosis [21,22]. In women of reproductive age, uterine cancers lead to infertility. These cancers are also associated with many deaths. Worldwide, more than 950,000 women are affected annually by cervical and endometrial cancers, and as a result of this, more than 400,000 will die [23,24,25].

#### 1.3.1. Uterine Fibroids

Uterine fibroids are also known as leiomyomas or uterine myomas, and they are the most common benign uterine tumors [26] that develop in pre-menopausal women and affect from 43% to 70% of women during their life [21]. The majority of myoma cases are asymptomatic, and are diagnosed coincidentally during routine pelvic examinations and ultrasound, the gold standard for diagnosis of myoma. On the other hand, 30–40% of the women affected show clinical symptoms, including pelvic pain and abnormal uterine bleeding [27].

These myomas are also linked to infertility, adverse pregnancy outcomes, and increased risk of pregnancy complications, such as miscarriage and preterm birth [26,28]. Traditionally, uterine fibroids are treated surgically, while conservative medical treatment focuses mainly on the myoma-related symptoms, such as bleeding and pain [29]. Recently, randomized controlled studies have demonstrated efficacy of the selective progesterone receptor modulator ulipristal acetate, which can alleviate bleeding and decrease myoma size [27].

The pathogenesis of myomas is multifactorial and includes genetic, epigenetic, hormonal, environmental, pro-inflammatory, and angiogenesis factors [30]. Patient ethnicity also has an important role, as shown by their higher incidence in African-American women [21]. There remains an unmet need for better understanding of the pathophysiology and for new options for treatments that can preserve the fertility of women with myomas.

#### 1.3.2. Adenomyosis

Adenomyosis is characterized by the presence of endometrial stromal and epithelial cells within the myometrium. This benign uterine disorder was traditionally diagnosed based on histological examination of hysterectomy specimens, the gold standard for diagnosis [31,32]. Nowadays, the improved imaging techniques (magnetic resonance imaging [MRI], transvaginal ultrasonography) allow diagnosis also in symptomatic women of reproductive age [31,32].

The symptoms of adenomyosis observed in the majority of women include heavy uterine bleeding, dysmenorrhea, and dyspareunia. Only a third of women are asymptomatic [31]. The estimated prevalence of adenomyosis is 20% in a general symptomatic population [22,31,33]. In up to 80% of cases, adenomyosis is associated with other benign uterine pathologies, and most commonly with myoma or endometriosis [34]. For decades adenomyosis has been known as endometriosis interna, but it is now clear that due to different clinical presentations and its specific pathogenesis, adenomyosis represents a distinct entity [31,35].

Adenomyosis also has a negative impact on fertility and pregnancy outcomes, and thus decreases the quality of life of these women [36]. As a chronic condition, it requires lifelong medical or surgical treatment [31]. Although there are no drugs approved for treatment of adenomyosis, several medications are used off-label to alleviate bleeding and pain, including nonsteroidal anti-inflammatory drugs, progestins, oral contraceptives, and gonadotropin-releasing hormone analogs [37]. The pathogenesis of adenomyosis is complex and elusive [31]. Currently there is no single theory that can explain all sub-types of adenomyosis, which calls for further research. The suboptimal diagnostic accuracy of two-dimensional and three-dimensional transvaginal ultrasonography [32] and the high costs of MRI also indicate that diagnostic biomarkers would be clinically relevant.

#### 1.3.3. Endometriosis

Endometriosis is a complex, estrogen-dependent, chronic inflammatory disease that is defined as the presence of endometrium-like tissue outside the uterine cavity [38,39,40]. This debilitating disease affects mainly women of reproductive age, and regresses after menopause [41]. Due to invasive diagnosis (i.e.; visualization by laparoscopy, histological examination of lesions), the exact prevalence of endometriosis is not known. Estimates show that up to 10% of premenopausal women and 35% to 50% of women with infertility, pelvic pain or both are affected [38,39,40].

Typical symptoms of endometriosis include dysmenorrhea, deep dyspareunia, chronic pelvic pain, and infertility, as well as dyschezia and dysuria [38,39,40]. As a result of the symptoms being generally nonspecific, there is an average delay of 6.7 years between onset of symptoms and surgical diagnosis of endometriosis [42]. Endometriosis thus significantly impairs the quality of life of patients [43,44].

Ectopic endometrial tissue can be found on the ovaries and in the pelvic peritoneum, and the rectovaginal septum, and also in other pelvic sites; this thus defines at least three different entities: ovarian, peritoneal and deep infiltrating endometriosis [45]. According to the American Society for Reproductive Medicine, endometriosis is classified into four stages, with these apparently not correlated with the intensity of symptoms [40,46].

Current management of endometriosis includes surgical removal of lesions and/or medical intervention [42] with nonsteroidal anti-inflammatory drugs and other analgesics, with off-label oral contraceptives and progestins as first line treatments of the symptoms [47]. Due to frequent recurrence, long-term pharmacological treatment is needed [39]. The pathogenesis of endometriosis is very complex and remains to be completely understood [41,48,49]. There is an urgent need for better treatment strategies and especially for the discovery of biomarkers for noninvasive diagnosis.

#### 1.3.4. Cervical Cancer

Cervical cancer (CC) is the fourth most-common cancer in women worldwide, estimates for 2018 indicate 569,847 new cases and the fourth highest cancer mortality rate, with 311,365 deaths [23]. In Europe and North America, it is the eighth and thirteenth most common cancer, respectively [23].

Cervical cancer develops in women of reproductive age and is most frequently diagnosed in women in their 30s and 40s [50]. Clinical symptoms of cervical cancer include vaginal bleeding, pelvic pain, dyspareunia, and postcoital bleeding, but these symptoms might not be present in patients with early cervical cancer [51]. The majority of cervical cancers (90%) are squamous cell (epidermoid) carcinomas, and 10% of cervical cancers are adenocarcinomas [51]. Cervical cytology together with human Papillomavirus (HPV) tests are commonly used for screening of cervical cancer, while diagnosis is based on colposcopy and directed biopsies [51]. Virtually all cervical cancers are associated with infection with HPV (reviewed by Faridi et al. [52]). Out of more than 40 HPV types that are sexually transmitted, 16 can cause cervical cancer [53]. HPV DNA has been detected in 99.7% of cervical cancer cases [50,54].

Cervical carcinogenesis is a complex process that includes integration of the HPV gene, with other changes as a consequence (e.g.; epigenetic modifications), and it culminates in uncontrolled cancer cell proliferation, evasion of tumor suppressors, tissue invasion, metastasis, angiogenesis, resistance to apoptosis, and cell immortality [50]. The treatment of cervical cancer includes surgery and chemoradiation therapies [51]. Early detection of cervical cancer improves prognosis, and thus biomarkers for noninvasive screening and diagnosis are urgently needed.

#### 1.3.5. Endometrial Cancer

Endometrial cancer (EC) is the sixth most-common female cancer worldwide, with estimates for 2018 of 382,069 new cases and 89,929 deaths [23]. In developed countries, the incidence of endometrial cancer continues to rise annually [55]. Diagnostic evaluation of endometrial cancer includes pelvic ultrasonography and endometrial sampling, as either biopsies or dilation and curettage [56]. Endometrial cancer is diagnosed mainly in post-menopausal women. However, about one fifth of women with endometrial cancer are premenopausal, and often want to preserve their fertility.

Sporadic endometrial cancer has been classified into two groups: estrogen-dependent type I, with an endometrioid histology, and which comprises 70–80% of all cases and develops from endometrial hyperplasia, and type II, which is considered estrogen-independent, and is more aggressive, has poor prognosis, develops de novo from atrophic endometrium [57,58], and comprises 20% of all cases. In addition to these sporadic cases, about 10% are hereditary [59,60], and are generally associated with hereditary nonpolyposis colorectal cancer (also known as Lynch syndrome) [61]. Nowadays, molecular classification provides the most relevant information about prognosis, with this based on integrated genomic, transcriptomic, and proteomic data.

Early stage endometrial cancer is treated by hysterectomy (or in exceptional cases with fertility sparing treatments), and is followed by adjuvant treatment of patients with higher stage disease, with increased risk of cancer recurrence, and with localized recurrent disease [55].

Currently there is an unmet need for targeted therapies of endometrial cancer [55], as well as for diagnostic biomarkers for screening high risk populations and prognostic biomarkers for the stratification of patients, to allow early detection and personalized treatment.

## 2. Objectives

With this review, we aim to address the following questions: Has metabolomics contributed to diagnosis or prognosis of uterine diseases to date? Has metabolomics contributed to deciphering the pathophysiology of uterine diseases to date?

## 3. Methods

### 3.1. Information Sources, Search Strategy, and Eligibility Criteria

**Figure 2:**
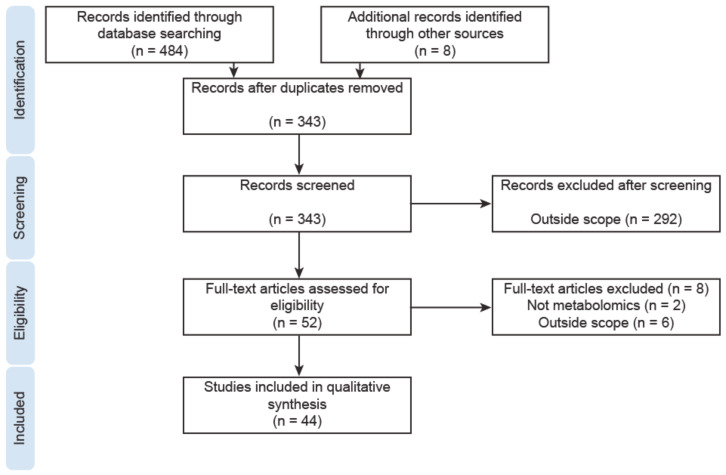
Study flow diagram.

We performed a systematic search of the literature in the PubMed, OVID (advanced search) and Scopus (advanced search) databases on 20 July 2020, using the search terms listed in Table 1. We focused on metabolomic studies performed in human tissue or physiological fluids. There was no restriction on publication date. Reports were retrieved, and the screening of titles and abstracts according to the inclusion and exclusion criteria (Table 2) was performed independently by authors TLR and JT. Disagreements were discussed and consensus was reached. Studies on metabolomics in reproductive medicine were reviewed relatively recently [62,63], and thus in the present analysis all reports on metabolomics in follicular fluid were excluded. Metabolomic studies performed in cell lines were out of the scope of this review, and so were also excluded. The screening process is shown in the study flow diagram (Figure 2).

### 3.2. Data Extraction

The reports selected were read in detail and all of the relevant data were extracted and collected in tables. The following data were gathered: authors and country of study; journal and year of publication; type of samples; storage of samples; methods used; study design; characteristics of case and control groups; major findings, including information about metabolites found at high/low levels for the particular pathology; and information about diagnostic/prognostic models/algorithms and their diagnostic/prognostic characteristics, if available. Reporting was performed under the guidance of the PRISMA diagnostic test accuracy checklist [64].

### 3.3. Assessment of Risk of Bias

**Figure 3:**
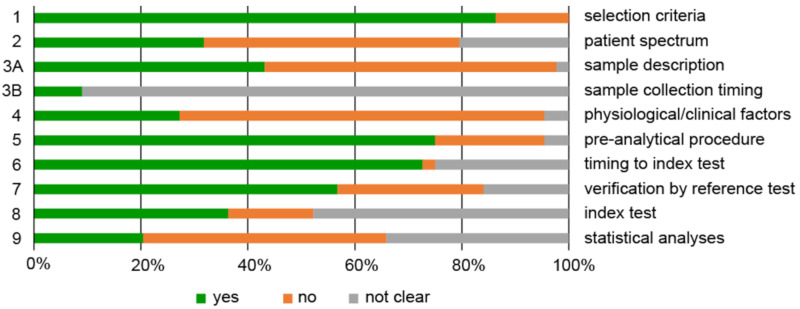
QUADOMICS scoring of all studies included. Proportion of studies with answers “yes”, “no”, or “not clear” to each of the selected signaling questions. Each signaling question is numbered on the left, and a short description of each question is given on the right. The detailed scoring is given in Supplementary Tables S2–S4.

The risk of bias and quality of individual diagnostic accuracy studies was assessed following the QUADOMICS tool, as an adaptation of QUADAS [65] that was designed especially for -omics studies [66] (Supplementary Table S1). This tool focuses on study design, patient selection, index test, reference standards, flow of timing, pre-analytical and analytical procedures, and statistical analysis [65]. For all studies included in this review, we responded to nine selected signaling questions to assess the quality of these studies (Supplementary Tables S1–S4, Figure 3).

## 4. Results

### 4.1. Study Selection

Our systematic search performed for the PubMed, OVID, and Scopus databases identified 484 articles in total. After screening of titles and abstracts according to the inclusion and exclusion criteria (Table 2), we excluded 149 duplicate papers and 292 papers that did not meet the criteria, such as studies in animal models, cell lines, nonuterine cancers, nonuterine benign pathologies, and follicular fluid, and studies focusing on transcriptomics (microRNAs), as well as review manuscripts, and studies dealing with MRI.

The final evaluation included 44 manuscripts, of which 36 were selected based on database searches, and eight were identified from reference lists. The selection process is depicted in the study flow diagram (Figure 2). The relevant data of these 44 studies are presented separately for individual diseases (Supplementary Tables S5–S8).

### 4.2. Study Characteristics

#### 4.2.1. Metabolomics in Uterine Fibroids and Endometriosis

The searches and further selection according to the inclusion and exclusion criteria identified one study on uterine leiomyoma and 17 studies on endometriosis. No metabolomics study has been performed in adenomyosis to date. Global metabolomics profiling in leiomyoma has been reported for tissue samples and corresponding myometrial specimens ( Supplementary Table S5) [67]. All leiomyomas showed dysregulation of 70 metabolites, and distinct metabolomics profiles were seen for different genetic subtypes of leiomyomas (i.e.; *FH*, *MED12*, *HMGA2*). This thus allowed stratification between subtypes and delineation of metabolic processes associated with development and growth of these benign tumors.

The majority of studies on endometriosis (Supplementary Table S6) examined metabolic profiles of blood samples (four serum, five plasma), five focused on eutopic/ectopic endometrium, two on peritoneal fluid, one on endometrial fluid, and one on urine. All of these studies investigated metabolic profiles with the aim to find diagnostic biomarkers of endometriosis in physiological fluids or tissue samples. Several diagnostic models have been reported with excellent diagnostic characteristics (Table 3). However, six of these diagnostic models distinguished between patients and healthy women only, which is not of direct clinical relevance. A diagnostic assay should define the patients with endometriosis within a group of symptomatic patients.

The targeted metabolomics studies focused mainly on lipids, and the nontargeted studies also identified mainly lipids, amino acids, and intermediary metabolites as the most important variables. High levels of lactate, 2-hydroxybutyrate, succinate, valine, and lysine and low levels of glucose, isoleucine, and arginine were reported by Dutta et al. and Jana et al. [68,69]. The highest diagnostic accuracies were reported for the plasma/serum metabolic signatures that separated healthy women from endometriosis patients [3,68,70]. For stratification between infertile women and endometriosis patients, a panel of serum metabolites was identified in a case/control discovery study, with a high AUC of 0.99: lactate, 2-hydroxybutyrate, succinate, and lysine [69]. A study based on nontargeted metabolomics in urine samples by Vicente-Munoz et al. reported significantly altered concentrations of 10 metabolites, but further principal component analysis failed to separate patients with endometriosis from healthy women undergoing sterilization [71]. On the other hand, a targeted metabolomics approach in peritoneal fluid revealed models with excellent diagnostic characteristics [72], and diagnostic potential was also seen for endometrial fluid [73]. Chagovets et al. recently performed nontargeted metabolomics in eutopic and ectopic endometrium of the same patients, and they reported differences mainly in phosphatidylcholines, and also sphingomyelins and phosphatidylethanolamines, which allowed separation between diseased and control tissue [74]. In eutopic endometrium, metabolomics studies revealed diagnostic algorithms that distinguished between infertile patients and patients with endometriosis with good diagnostic accuracies included phosphatidic acid, phosphatidylcholines, phosphatidylserine, lysophosphatidylethanolamine, uric acid, and hypoxanthine [75,76].

**Table 3:** Selection of studies that reported diagnostic and prognostic models potentially relevant for clinical practice.

**Sample**	**Method**	**Findings/Models**	**Reference**
**Uterine fibroids**			
tissue samples	LC-MS/MS	Leiomyomas/myometrium: 70 metabolites dysregulated; ↓ homocarnosine, haeme, biliverdin, stratification between FH and MED12 subtype	[67]
**Endometriosis**			
serum	^1^H-NMR	↑ lactate, 2-hydroxybutyrate, succinate, Lys, glcerophosphocholine, citric acid, pyruvate, adipic acid; ↓ Ile, Leu, Arg, Asp, Ala, Glu, creatine; **IW/E AUC: 0.99,** SEN: 100%**,** SP: 91.6%	[69]
plasma	LC-MS/MS	↑ lauroylcarnitine, oleylcarnitine, myristoylcarnitine, long-chain acylcarnitines; ↓ trimethylamine-N-oxide; **CP/E**: SEN: 81.8%, SP: 88.9%, PPV: 75%	[77]
peritoneal fluid	ESI-MS/MS	**HW/OE** (C0/PCae C36:0, PCaa C30:0/PCae C32:2, age) **AUC**: 0.94, SEN: 82.8%, SP: 94.4%	[72]
endometrial fluid	LC-MS/MS	↓ sphingolipids, glycerolipids; ↑ mono or polyunsaturated TAG; **CW/OE** (model of 123 metabolites): SEN: 58.3%, SP: 100%	[73]
eutopic endometrium	LC-MS	**IW/E** (PC (18:1/22:6), PC (20:1/14:1), PC (20:3/20:4), PS (20:3/23:1), PA (25:5/22:6)); **AUC: 0.87**, SEN: 90.5%, SP: 75.0%	[75]
eutopic endometrium	LC-MS	**IW/E** (uric acid, hypoxanthine, lysophosphatidylethanolamine); **AUC: 0.87**, SEN: 66.7%, SP: 90.0%	[76]
**Cervical cancer**			
plasma	LC-MS	**CR/SD:** (Val + Trp) **AUC: 0.94**, SEN: 87%, SP: 80%; **CR + PR/SD:** (Trp) **AUC: 0.82**	[78]
plasma	LC-MS/MS	**HW/CC:** (bilirubin, LysoPC (17:0), n-oleoyl Thr, 12-hydroxydodecanoic acid, tetracosahexaenoic acid); **AUC: 0.99**, SEN: 98%, SP: 99%	[79]
plasma	LC-MS	phthalic acid, D-maltose, PG (12:0/13:0), LacCer (d18:1/16:0), PC (15:0/16:09); **CC before/poor prognosis**: **AUC: 0.97**, SEN: 94%**,** SP: 87%; **CC before/good prognosis**: **AUC: 0.97**, SEN: 92%**,** SP: 89%	[80]
**Endometrial cancer**			
plasma	LC-MS/MS	**BD/EC** (C16/PCae C40:1, Pro/Tyr, PCaa C42:0/PCae C44:5); **AUC: 0.84**, SEN: 85.3%, SP: 69.2% **MI-/MI+** (SMOH C14:1/SMOH C24:1, PCaa C40:2/PCaa C42:6); **AUC: 0.86**, SEN: 81.3%, SP: 86.4% **LVI-/LVI+** (PCaa C34:4/PCae C38:3, C16:2/PCaa C38:1); **AUC: 0.94**, SEN: 88.9%, SP: 84.3%	[81]
plasma	LC-MS/MS	**EC long/short survival** (MetSO, serotonin, spermine, C3-OH, PCaa C36:5, SM C20:2, spermidine, C4:1, lyso PCaa C18:2, lysoPCaa C24:0, Asp, dimethylarginin, hexose, PCae C30:1); **AUC: 0.965**	[82]
serum	GC-MS	**EC type 1/type 2:** ↓ lactic acid, cystine, Ser, malate, Glu, homocysteine; ↑ progesterone; **Accuracy: 0.93**, SEN: 96%, SP: 86%	[83]
serum	LC-MS/MS	**C/EC** (spermine, isovalerate, glycylvaline, gamma-glutamyl-2-aminobutyrate); **AUC: 0.92** **R/NR** (2-oleoylgycerol, TAG 42:2-FA12:0); **AUC: 0.90**	[84]
cervico-vaginal fluid	^1^H-NMR	**CP/EC** (phosphocholine, malate, Asp); **Accuracy: 0.78**, SEN: 75%, SP: 80%	[85]

Legend: AUC, area under the curve; BD, benign diseases; BT, before treatment; CC, cervical cancer; CIN, cervical intraepithelial neoplasia; CP, control patients; CR, patients with complete response to chemotherapy; CW, control women; E, endometriosis, EC, endometrial cancer; FA, fatty acid; IW, infertile women; NR, non-recurrent; OE, ovarian endometriosis; PPV, positive predictive value; PR, patients with partial response to chemotherapy; R, recurrent; MI, myometrial invasion; LVI, lymphovascular invasion; TAG, triacylglycerol; PA, phosphatidic acid; PC, phosphatidylcholine; PS, phosphatidylserine; PCae, PCaa, glycerophospholipids; SD, patients with stable disease; SEN, sensitivity; SP, specificity; SMOH, hydroxysphingomyelin. Only studies that reached a QUADOMICS score (no/not clear ≤60%) were included. All models need further validation.

#### 4.2.2. Metabolomics in Cervical and Endometrial Cancers

We identified 12 studies on CC (Supplementary Table S7) and 14 studies on EC (Supplementary Table S8). The majority of these studies evaluated metabolic profiles in blood samples (six serum, ten plasma), with six studies in endometrial or cervical tissue samples, and two each in cervico-vaginal fluid and urine samples.

Studies in CC were performed with the aim to identify novel options for early diagnosis (11 studies) or to identify predictive (one study) or prognostic (one study) models. Metabolomics profiles of blood, urine, cervico-vaginal lavage and tissue samples from cancer patients were compared to those of healthy women and patients with benign uterine pathologies (e.g.; cervicitis, precancerous cervical intraepithelial neoplasia [CIN]) [79,86,87,88,89,90,91]. Algorithms with excellent diagnostic characteristics were reported for plasma samples (Table 3) [79,80,90]. Yin et al. [90] reported the discovery and validation of a model that included the combination of four metabolites, two phosphatidylcholines and two lysophosphatidylcholines, and provided discrimination between CC patients and patients with uterine fibroids, with sensitivity of 93%, specificity of 91%, and AUC of 0.97. A diagnostic algorithm that included bilirubin, lysophosphatidylcholine, and three long-chain fatty-acid derivatives was reported by Yang et al. [79] that distinguished CC patients from healthy controls with AUC of 0.99. Another diagnostic algorithm by Khan et al. based on metabolic profiling in plasma samples showed good diagnostic characteristics for stratification between healthy women and patients with CIN (AUC of 0.82), and also between healthy women and patients with CC (AUC of 0.83) [87]. On the other hand, Zhou et al. [80] reported prognostic algorithms with AUC > 0.9 based on comparison of the plasma metabolomes from CC patients with and without recurrence. Studies in cervico-vaginal lavage have also shown that metabolites separate between HPV-negative healthy pre-menopausal women and patients with low-grade squamous intraepithelial lesions and high-grade squamous intraepithelial lesions, with AUC > 0.8, and also between healthy women and patients with invasive cervical cancer, with AUC of 0.92 [88]. Two studies in cervical tissue samples revealed that 17 metabolites can be used to differentiate between normal cervical tissue and CIN or squamous CC, and also between CIN and squamous CC [86]. Finally, Tokareva et al. [89] performed shotgun metabolomics and reported that nonpolar glycerolipids distinguish between border cervical tissue and CC tissue with AUC of 0.95.

The metabolomics studies in EC were performed with the aims to identify diagnostic biomarkers (seven studies) or diagnostic and/or prognostic biomarkers (three studies), or to contribute to better understanding of the pathogenesis (four studies). Several dysregulated pathways were identified, including those for lipid biosynthesis and metabolism (Table 3). Alterations of a series of lipid metabolites were reported for tissue and blood samples, including glycerophospholipids, lysophospholipids, sphingolipids, fatty acids, acylcarnitines, prostaglandin metabolites, and steroid hormones [81,83,84,92,93,94,95,96]. In serum and plasma samples, glycerophospholipids in combination with other metabolites showed diagnostic and prognostic potential [81,82,96], while changes in the profiles of these metabolites in EC tissue were associated with tumor progression [93]. Also, sphingolipids [81,84] and acylcarnitines [81,82,84,96] revealed diagnostic and prognostic potential. Good diagnostic accuracy to distinguish between EC and healthy women was reported for a model that included serum lactic acid, homocysteine, 3-hydroxybutyrate, linoleic acid, stearic acid, myristic acid, threonine, valine, and progesterone [83]. Good characteristics for stratification between control patients and EC patients was shown for a model that included plasma levels of single metabolites and ratios between different metabolites, mainly for phosphatidylcholines and sphingomyelins, but also for proline and tyrosine (AUC, 0.84) [81], as well as for a model that included histidine, isoleucine, valine, and proline [97]. The best prognostic models with AUC of 0.94 and 0.97 distinguished between patients with long and short survival, respectively. These models included a series of plasma metabolites, as phosphatidylcholines, acylcarnitines, lysophosphatidylcholines, aspartic acid, and several low molecular weight metabolites (i.e.; serotonin, spermine, spermidine, dimethylarginine, hexose, methionine sulfoxide) [82]. Our recent study revealed that alterations in metabolic profiles allow pre-operative prognosis of lymphovascular invasion, which is considered one of the main prognostic factors [81]. Combinations of a ratio between two phosphocholines and a ratio between acylcarnitine and phosphocholine showed excellent characteristics, with AUC of 0.94 [81]. Audet-Delage et al. compared metabolomics profiles between recurrent and nonrecurrent patients and constructed a model that included triacylglycerol and 2-oleylglycerol, with AUC of 0.9 [84]. Metabolomic profiling in tissue samples indicated that prostaglandin metabolism [98] and endocannabinoid signaling are affected in endometrial cancer [93,99]. Several steroids were shown to be associated with recurrence of type 1 EC, including estrone sulfate [95] and the bile acids taurodeoxycholate, glycodeoxycholate, and taurocholate [84].

These studies thus demonstrate the great value of metabolomics for the discovery of diagnostic and prognostic biomarkers of CC and EC, and the possibility for clinical applications of the biomarker panels or algorithms identified.

### 4.3. Quality of the Selected Studies

The quality of the studies included was evaluated systematically according to the QUADOMICS tool (Supplementary Tables S1–S4; Figure 3). We assessed study design and pre-analytical, analytical, and post-analytical bias of the selected studies. The criteria for the selection of patients (question 1) were usually well described. The majority of studies did not reproduce the real clinical setting, as they compared healthy women with patients, which is quite common for discovery phase studies (question 2). The assessment of pre-analytical bias (question 3A, B) revealed that more than half of these studies failed to provide appropriate descriptions of the samples, including the procedures for how they were obtained and processed (e.g.; type of blood tubes, centrifugation time). Furthermore, the majority of the studies did not report information about time of sample collection, time between taking of blood and its centrifugation, time between sample acquisition and storage, and number of freeze/thaw cycles. More than 70% of studies also did not provide information on clinical and physiological factors (question 4) that can affect metabolomics data (e.g.; fasting status, body mass index [BMI], menopausal status, menstrual phase). In 18 of the 28 studies that evaluated metabolomic signatures in serum, plasma or urine samples, case and control patients were fasted before sample collection. Two studies were performed including nonfasting patients [86,90]. For the remaining eight studies, there were no data about fasting status. The majority of the studies reported detailed descriptions on sample storage and metabolite extraction (question 5). Almost all of the samples were stored at −80 °C, with the exception of one study, where serum samples were stored at −60 °C [91], and two studies where serum [69] and plasma [70] were stored at −20 °C. For several studies there were no data reported about sample collection and storage [89,90,100,101]. The time between the reference standard and the index test (metabolomics) was not known or was not clear for 27% of all of the studies (question 6), while in 25% of the studies, the verification by reference test was not performed in all patients (question 7).

We next evaluated analytical bias, where the majority of studies had incomplete reports on the metabolomics analysis (question 8). This mainly involved a lack of reporting for the descriptions of sample randomization for MS-based metabolomics and for the use and type of quality control samples. The analysis for post-analytical bias revealed that only 10 studies (22%) applied and described the statistical analysis in sufficient detail (question 9). The majority of studies failed to report information on sample to sample normalization, and data transformation and scaling, and in some cases, also on cross-validation methods for the generation of the diagnostic and prognostic models.

In general, by comparing QUADOMICS scores for different pathologies (Supplementary Figure S1), we noticed that studies in endometriosis had the highest (58.2%) mean score (i.e.; mean percentage of scoring “yes” for all studies investigating a specific pathology) and those in cervical cancer the lowest mean score (37.5%).

## 5. Discussion

### 5.1. Main Findings

#### 5.1.1. Contribution to Identification of Diagnostic and Prognostic Biomarkers

This literature review revealed that over the last decade, metabolomics has contributed to the identification of biomarker candidates for diagnosis of endometriosis and CC, and for prognosis of CC and EC. Metabolomics studies might also contribute to pinpointing diagnostic and prognostic biomarkers in adenomyosis and uterine fibroids, which calls for well-designed metabolomics studies. Options for noninvasive or semi-invasive diagnosis would allow earlier diagnosis, while options for pre-operative stratification would contribute to tailored treatments, which is of outmost importance especially for patients with endometriosis and CC and EC. The majority of metabolites and the corresponding models/algorithms identified to date represent biomarker candidates that need to be validated in further studies.

Although models that include combinations of biomarkers and clinical data are currently in clinical use, to the best of our knowledge combinations of different -omics data together with clinical data have not been reported yet. This approach might lead to construction of models/algorithms with much better diagnostic/prognostic characteristics, which we strive to achieve by combining metabolomics, proteomics, and clinical data in the current BioEndoCar project (www.bioendocar.eu). Such efforts are needed for other uterine diseases as well.

#### 5.1.2. Contribution to Deciphering Pathophysiologies

**Figure 4:**
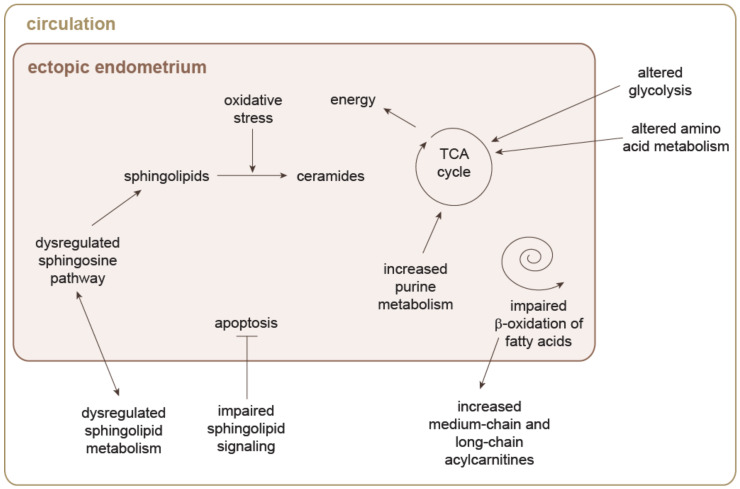
Speculative model of alterations in metabolic pathways in endometriosis, based on the identified biomarkers for endometriosis. Interactions between pathways and interactions between the endometrium tissue and the circulation were not experimentally validated in the studies included into this review. Arrows do not necessarily imply causal relationships.

For uterine fibroids, there are no sufficient metabolomics data, because to date, only one study was conducted in tissues [67]. The data availability for endometriosis are a lot better (Supplementary Table S6). Studies in serum and plasma showed dysregulated sphingolipid metabolism [102] and sphingomyelin signaling [3], which might lead to suppression of apoptosis, increased oxidative stress, and impaired plasma-membrane composition (Figure 4). Increased levels of medium-chain and long-chain acylcarnitines [77] might indicate impaired or less efficient β-oxidation of fatty acids [3]. To compensate for the less efficient energy generation by β-oxidation, it can be speculated that the altered amino acid metabolism and increased glycolysis in serum [68,69] will have a role. This hypothesis is supported by the metabolite changes observed in eutopic endometrium. The levels of certain amino acids were lower [68], while purine metabolism was increased [76]. The metabolites of these pathways might fuel the tricarboxylic acid cycle, to address the energy demands of the cells.

Some studies reported lower levels of sphingomyelins in peritoneal fluid [72,73,103]. Taken together with dysregulation of the sphingosine pathway in ectopic endometrium [104], this observation implies greater oxidative stress in endometriosis lesions, because oxidative stress promotes the conversion of sphingomyelins to ceramides [105]. Ceramides, in turn, were high in peritoneal fluid [73], which might lead to increased formation of sphingosine-1-phosphate, which is involved in angiogenesis and cell migration and apoptosis [104].

For CC, several studies showed changed levels of amino acids in plasma or serum, although in contradictory directions. Hasim et al. reported lower levels of isoleucine, valine, alanine, glutamine, histidine, and glycine in plasma [106,107], while Ye et al. reported higher levels of glycine and alanine in serum [91]. Altered levels of amino acids in blood are known to occur in cancer patients, as these metabolites are the building blocks for tumor biomass [2]. Higher levels of lactate in cervical tumor tissues [86] might support tumor growth. Tumor cells are known to generally use aerobic glycolysis to generate ATP, which is also known as the Warburg effect [2], and lactate is the end product of this pathway. Lactate is secreted by tumor cells and has angiogenic effects [2,108], which further supports the tumor growth. Many other studies have reported changes to single sets of metabolites (especially single lipids) in patients with CC. However, these changes are too widespread on the metabolic pathway map to be interrelated.

**Figure 5:**
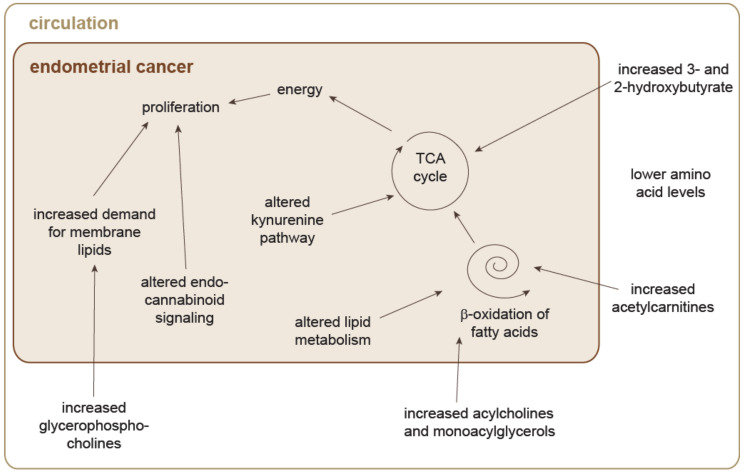
Speculative model of alterations in metabolic pathways in endometrial cancer based on identified biomarkers for endometrial cancer. Interactions between pathways and interactions between the cancer tissue and the circulation were not experimentally validated in the studies included into this review. Arrows do not necessarily imply causal relationships.

For EC, metabolomics profiling has revealed specific signatures compared to normal endometrium, and has also allowed discrimination between invasive and noninvasive tumors and the histological grades of tumors [99]. Serum from patients with EC contained higher levels of acylcholines, monoacylglycerols, and acylcarnitines [81,84], as well as higher levels of 3-hydroxybutyrate and 2-hydroxybutyrate [96]. Monoacylglycerols and acylcarnitines might be used for β-oxidation in the liver, which yields 3-hydroxybutyrate and 2-hydroxybutyrate (Figure 5). These intermediates might be used as energy sources by the tumor [109]. The levels of some amino acids were also seen to be lower in the circulation of patients with EC compared to controls [83,97]. Similar to endometriosis and CC, this can be explained by increased amino acid metabolism in the tumor to support its energy demands for cell proliferation. For the tissue, changes were described mainly for lipid metabolism, endocannabinoid signaling, purine metabolism, and the kynurenine pathway [93,98,99]. Higher levels of glycerophosphocholines in cancer tissue and cervico-vaginal fluid from patients with EC [85,93] imply increased demand for membrane lipids to support tumor proliferation [85]. Alterations in central carbon metabolism were deduced from the metabolic changes in serum from patients with EC [83], although these were not shown directly in tumor tissues. However, it is expected that changes in glycolysis occur, because some of the enzymes involved in glycolysis have been suggested as biomarkers for EC, namely pyruvate kinase and phosphoglycerate kinase [110]. Their higher expression in malignant cells underlines their role in ATP generation in glycolysis.

However, most of the studies included in this review were conducted to identify biomarkers, and were not intended to help to explain the underlying biology [111]. Elucidation of underlying disease mechanisms requires a combination of different -omics technologies in mechanistic study designs. Therefore, these reflections on the underlying disease-related mechanisms can only be speculative.

### 5.2. Limitations of the Included Studies

The human metabolome is extremely dynamic and is influenced by a vast number of factors, from feeding status, hydration, circadian rhythm, and life-style (e.g.; smoking, alcohol consumption, exercise), to comorbidities [6]. Therefore, confounders in metabolomics studies need to be tightly controlled [112] and standard operating procedures are a prerequisite [113]. Furthermore, it is essential that patients enrolled in metabolomics studies are characterized in detail with regard to demographic, clinical, and life-style data. For reproducibility of findings in metabolomics, it is of vital importance to standardize metabolite extraction and measurements, and to provide detailed descriptions of all aspects related to sample processing and metabolite measurement. As metabolomics analyses deliver information-rich datasets, appropriate data pre-processing (including sample to sample normalization, data transformation and scaling) and statistical methods (such as cross validation) need to be applied [6], especially in the context of biomarker identification studies [111].

The control groups were often not chosen from the target population, which is quite common for discovery phase studies. Yet, this might diminish the discriminative power of the identified biomarkers in the target population, i.e.; symptomatic women with or without the disease. Frequent lack of reporting confounding factors, especially age, BMI, menstrual phase, menopausal status, other comorbidities, as well as fasting status, implies that these studies did not take into account the impact of confounding factors on the metabolome, and thus, the models obtained were not adjusted for these factors. Failure in reporting sufficient details on sample processing, metabolite extraction, and parameters of the metabolomics measurements prevents replication of the findings from any particular study, and prohibits the application of any newly developed index test based on metabolomics to other settings. The usage and provision of information on quality control samples is of vital importance. Quality control samples enable the judgement of the quality of metabolomics measurements in general. Also, they are crucial for nontargeted metabolomics, because they enable signal correction to reduce analytical variation [114].

In the absence of sufficient information on data preprocessing, biomarker selection, and performance evaluation of the obtained models, the reported models can be questioned. Performance evaluation of the models by cross-validation should ideally split the dataset into a training and a test set, where the training set is used to build the model, and the test set is used to determine the true performance of the model [111].

Despite successful statistical validation of the models obtained, any final model for novel biomarkers needs to be validated experimentally. Only one study collected a second cohort to validate the biomarkers identified in the first cohort [90], further underlining that all included studies were in the discovery phase of biomarker identification.

### 5.3. Challenges and Future Perspectives

The identification of diagnostic biomarkers for adenomyosis, endometriosis and cervical cancer, and for prognostic and predictive biomarkers for cervical and endometrial cancers, is an important and necessary task, and metabolomics is a valuable tool to address this need. These diseases endanger the reproductive status of women, and therefore early diagnosis and treatment is of paramount importance. The studies included in this review analyzed liquid biomaterials, including plasma, serum, urine, and cervico-vaginal and endometrial fluids, and also tissue samples. Cervico-vaginal and endometrial fluids and cervical tissue represent interesting sources for diagnostic and prognostic biomarkers, while blood- and urine-derived biomarkers allow development of noninvasive tests, and thus show greater potential.

However, even apparently similar fluids such as plasma and serum differ widely in terms of their metabolite compositions. Plasma is prepared from freshly drawn blood by centrifugation, while serum is obtained after coagulation. Thus, the serum metabolome is affected by the metabolism of the blood cells, as well as by different clotting times [115,116]. There were significantly different metabolite concentrations between serum and plasma for 46% of 216 metabolites analyzed [115]. Any biomarkers identified in serum for a specific uterine disease might not necessarily be biomarkers for the same disease in plasma.

The selected studies reported diagnostic and prognostic models with good characteristics. However, none of the biomarker models obtained has been experimentally validated. To do so, replicate samples from an independently collected validation cohort need to be analyzed in independent laboratories, ideally using two different metabolomics methods [111]. Typically, biomarker discovery studies include a small number of participants compared to the size of the expected target population [111]. Discovery studies are usually conducted in a single study center, which makes it easier to control confounding factors and to adhere to sample collection protocols. To follow the guidelines of Xia et al. [111], further clinical validation on a larger scale is necessary after successful validation of a biomarker panel at one study center. For this, multicenter studies need to collect large cohorts that should cover all ethnicities and possible confounding factors, such as age, BMI, and life-style habits. The selection of an appropriate control group is also of the utmost importance, to allow discrimination between patients with the disease from patients with the same set of symptoms but without the disease. While nontargeted metabolomics is an excellent method for biomarker discovery, the biomarkers identified should be validated by a targeted metabolomics assay, which ideally contains the most promising candidate metabolites and delivers absolute concentrations, which is preferable for diagnostic and prognostic tests [111]. Further, metabolomics has potential to replace current diagnostic histology. Shot gun metabolomics (i.e.; flow injection metabolomics) differentiated border cervical and cervical cancer tissues, and is faster than histological examination, thus having great potential for future molecular diagnostics [89].

Future research will have to address the question whether metabolomics allows differentiation between adenomyosis, endometriosis, and uterine fibroids, as well as between endometrial cancer and cervical cancer. Possible co-occurrence of uterine fibroids, adenomyosis, and endometriosis [34] brings additional challenge to the development of diagnostic biomarkers for these diseases. Three of the studies included into this review approached the discrimination of different gynecological cancers with inconclusive results. Woo et al. were not able to distinguish between ovarian cancer and cervical cancer [117]. Troisi et al. observed that the serum metabolome of patients with endometrial cancer differed from that of patients with ovarian cancer [83]. However, a model based on phosphocholine, malate, and asparagine established by Cheng et al. had no discriminatory power to distinguish between patients with endometrial cancer and cervical cancer [85]. Thus, further research in this field is needed.

Future research will have to unravel whether metabolic biomarkers have the power to differentiate between cervical cancer and endometrial cancer, and between other cancer types. This is essential, because all cancer types change their metabolism to higher glucose dependency and higher amino acid consumption [118], among other metabolic changes. Therefore, any blood-based biomarker or biomarker panel that is specific for cervical cancer or endometrial cancer has to be validated carefully, to ensure that this marker or marker panel is not a marker for other types of cancer. This robust differentiation between several diseases by biomarkers is currently one of the biggest challenges.

## 6. Concluding Remarks

This review set out to answer the first question of whether metabolomics has so far contributed to the diagnosis or prognosis of uterine diseases. For endometriosis and cervical and endometrial cancers, several biomarker panels with good performances have been suggested. Some diagnostic models for endometriosis have great potential for translation into the clinic. Also, in cervical and endometrial cancer, promising diagnostic and prognostic models have been generated. For uterine fibroids, just one study to date provides room for further investigation. Technological advancements such as nanoflow liquid chromatography or ultrahigh resolution mass spectrometry will enhance precision and reproducibility as well as mass accuracy in future. However, high analytical power is wasted if sample associated data is lacking. There is an urgent need for better description of the samples and the physiological and clinical factors, as well as the metabolomics measurements and statistical data evaluation. Provision of detailed descriptions are of vital importance, to allow replication and better reproducibility of these studies.

The second question addressed concerned the contribution of metabolomics to deciphering the pathophysiology of uterine diseases. Differences in metabolite levels determined by case/control studies are descriptive in nature, and do not allow conclusions on the underlying pathophysiological mechanisms. Unravelling disease mechanisms not only requires the complete metabolomics characterization of several biospecimens of diseased patients (e.g.; blood-based fluids, diseased and healthy tissue samples, fluids surrounding affected tissues), but also the combination with other -omics technologies. Indeed, complete characterization of several patient biofluids would have been included in this review, but they have yet to be performed. Thus, the contribution of metabolomics to deciphering the pathophysiology of uterine diseases remains speculative to date.

In general, the large numbers of investigations into biomarker discovery in uterine diseases by metabolomics demonstrate that the potential of metabolomics has been recognized in the field of gynecology and reproduction. However, future studies are needed for the appropriate validation of biomarkers identified, and to ensure their specificity. After these requirements are met, it is expected that algorithms based on metabolites will contribute to better diagnostic options for endometriosis and diagnostic and prognostic alternatives for cervical cancer and endometrial cancer.

## Figures and Tables

**Table 1:** Search strategy for identification of manuscripts in Pubmed, OVID, and Scopus.

Search Query	Search Results	Selected Manuscripts	Additional Manuscripts	Included
(myoma uteri) OR (uterine myoma) OR (uterine myomas) OR leiomyoma OR leiomyomas OR (uterine fibroid) OR (uterine fibroids) OR (uterus myomatosus) AND metabolomic AND (English[Language]) NOT review	8 (Pubmed) 3 (OVID) 12 (Scopus)	1 (Pubmed) 1 * (OVID) 0 (Scopus)	0	1
adenomyosis OR (endometriosis interna) AND metabolomic AND (English[Language]) NOT review	1 (Pubmed) 0 (OVID) 1 (Scopus)	0 (Pubmed) 0 (OVID) 0 (Scopus)	0	0
endometriosis AND (metabolomic OR metabolomics) AND (English[Language]) NOT review	31 (Pubmed) 21 (OVID) 38 (Scopus)	13 (Pubmed) 10 * (OVID) 4 */1 (Scopus)	3	17
(cervical cancer) OR (cervix cancer) OR (cervical carcinoma) AND (metabolomic OR metabolomics) AND English[Language]) NOT review	81 (Pubmed) 21 (OVID) 96 (Scopus)	9 (Pubmed) 5 * (OVID) 1 * (Scopus)	3	12
(endometrial cancer) OR (uterine cancer) OR (endometrial carcinoma) OR (uterine carcinoma) AND metabolomic AND (English[Language]) NOT review	69 (Pubmed) 10 (OVID) 92 (Scopus)	12 (Pubmed) 8 * (OVID) 2 * (Scopus)	2	14
Total	190 (Pubmed) 55 (OVID) 239 (Scopus)	35 (Pubmed) 24 * (OVID) 1/7 * (Scopus)	8	44

* duplicate manuscripts.

**Table 2:** Inclusion and exclusion criteria.

**Inclusion criteria**	research papers, papers in English, studies in humans, tissue or physiological fluids, except follicular fluid *
**Exclusion criteria**	review papers, papers in other languages, studies in animals, follicular fluid, studies including only unidentified metabolites, studies focusing on fertility *, studies in cell culture **

* Studies in follicular fluid focusing on infertility and reports on metabolomics in reproductive medicine have been reviewed relatively recently by Bracewell-Milnes et al. [62] and Siristatidis et al. [63]. ** These studies were out of the scope of our review.

## Supplementary Materials

The following are available online at https://www.mdpi.com/2075-4426/10/4/294/s1, Table S1: Selected signaling questions to assess the quality of the selected manuscripts, Table S2: QUADOMICS scoring of the included studies for uterine fibrosis and endometriosis, Table S3: QUADOMICS scoring of the included studies for cervical cancer, Table S4: QUADOMICS scoring of the included studies for endometrial cancer, Figure S1: QUADOMICS scoring of the included studies for endometriosis, cervical cancer, and endometrial cancer separately, Table S5: Metabolomics in uterine fibrosis, Table S6: Metabolomics in endometriosis, Table S7: Metabolomics in cervical cancer, Table S8: Metabolomics in endometrial cancer, Table S9: PRISMA 2009 checklist.
