# Supplementary material for: Metabolomics for Diagnosis and Prognosis of Uterine Diseases? A Systematic Review
Source: J Pers Med. 2020 Dec 21;10(4):294. doi: 10.3390/jpm10040294 (PMC7767462; doi:10.3390/jpm10040294)
Supplement: Supplementary file 1 [file jpm-10-00294-s001.pdf]

Metabolomics for diagnosis and prognosis of uterine diseases?

A systematic review

Janina Tokarz[1], Jerzy Adamski[1,2,3,4] and Tea Lanišnik Rižner[5]*

[1] Helmholtz Zentrum München, German Research Centre for Environmental Health, Research Unit Molecular Endocrinology and Metabolism, Ingolstaedter Landstrasse 1, 85764 Neuherberg, Germany
[2] German Centre for Diabetes Research, Ingolstaedter Landstrasse 1, 85764 Neuherberg, Germany
[3] Lehrstuhl für Experimentelle Genetik, Technische Universität München, Freising-Weihenstephan, Germany
[4] Department of Biochemistry, Yong Loo Lin School of Medicine, National University of Singapore, Singapore, Singapore
[5] Institute of Biochemistry, Faculty of Medicine, University of Ljubljana, Vrazov trg 2, 1000 Ljubljana, Slovenia

Supplementary Materials:

Supplementary Table S1: Selected signaling questions to assess the quality of the selected manuscripts.

Supplementary Table S2: QUADOMICS scoring of the included studies for uterine fibrosis and endometriosis.

Supplementary Table S3: QUADOMICS scoring of the included studies for cervical cancer.

Supplementary Table S4: QUADOMICS scoring of the included studies for endometrial cancer.

Supplementary Figure S1: QUADOMICS scoring of the included studies for endometriosis, cervical cancer, and endometrial cancer separately.

Supplementary Table S5: Metabolomics in uterine fibrosis.

Supplementary Table S6: Metabolomics in endometriosis.

Supplementary Table S7: Metabolomics in cervical cancer.

Supplementary Table S8: Metabolomics in endometrial cancer.

Supplementary Table S9: PRISMA 2009 checklist.

Supplementary Table S1: Selected signaling questions to assess the quality of the selected manuscripts.

	**QUADOMICS signaling questions**	Yes, No, Not clear (NC)
**1**	**Was selection criteria clearly described?**<br>*Inclusion/exclusion criteria,*<br>*detailed information on sources of samples*<br>*(flow diagram not needed)*	
**2**	**Was the spectrum of patients representative?**<br>*Target population that would need diagnostic or prognostic test.*	
**3A**	**Was the type of sample fully described?**<br>*Type of sample (serum, plasma, tissue sample, etc.)*<br>*(for plasma: EDTA, heparin, citrate), time before centrifugation for serum!*<br>*centrifugation time and g (not rpm)*<br>*how were tissue sample obtained*	
**3B**	**Was the collection procedure of sample fully described?**<br>*time of sample collection (morning, during the day, …)*<br>*time between blood flow and centrifugation (delay in processing)*<br>*time between sample acquisition and storage*<br>*freeze-thaw cycles*<br><br>*for tissues:*<br>*time between collection and freezing*	
**4**	**Were the procedures of biological sample collection with respect to clinical factors described with enough detail?**<br>*Clinical and physiological factors?*<br>*Age, fasting status, BMI, menstrual phase, menopausal status)*	
**5**	**Were handling and pre-analytical procedures reported in sufficient detail and similar for the whole group?**<br>*If differences in procedures were reported, was their effect on the results assessed?*<br>*Detailed description of pre-analytical procedures: temperature of storage, procedure of metabolite extraction.*	
**6**	**Is the time between the reference standard and the index test short enough to guarantee that the target condition did not change between the two tests?**<br>*Samples are usually obtained before or during surgery, which is considered a reference standard.*	
**7**	**Did the whole sample or a random selection of the sample receive verification using a reference standard of diagnosis?**	

	In the case/control studies healthy controls did not undergo surgical treatment.	
**8**	**Was the execution of the index test described in sufficient detail to permit replication of the test?**<br>*Metabolomics analysis: description of the MS or NMR* ***method****,* ***control procedures****, (calibration and randomization only for MS)*	
**9**	**Was statistical analysis of the index test described in sufficient detail?**<br>*Statistical methods, reproducibility assessment, normalization, transformation and cross-validation (leave-one-out, bootstrap, jackknife and permutation tests, independent training and test set)*<br>*Validation test performed:* ***yes****/no OR*<br>*Other approaches for overfitting:* ***yes****/no*	

Supplementary Table S2: QUADOMICS scoring of the included studies for uterine fibrosis and endometriosis.

Study/QUADOMICS	1	2	3A	3B	4	5	6	7	8	9	comments
**Uterine fibroids (1)**											
Heinonen 2017 tissue	no	yes	no	NC$	no*	no	yes	yes	NC	yes	*no clinical data, $no information on daytime of sample acquisition, timing of sample processing, and freeze-thaw cycles
**Endometriosis (17)**											
Vouk 2012 plasma	yes	no*	yes	yes	yes	no**	yes	yes	yes	NC#	*HW, **Metabolite extraction, # no info on transformation, scaling, cross validation
Dutta 2012 serum	yes	no*	no**	NC$	no***	no****	NC	yes	yes	yes	*HW. **time to centrifugation, ***fasting **rpm, $no information on daytime of sample acquisition, timing of sample processing
Jana 2013 serum	yes	yes	no*	NC$	no**	yes	NC	NC	yes	yes	*time to centrifugation,**type and stage of disease, $no information on timing of sample processing
Lee 2014 serum, PF, tissue	yes	yes	NC	NC$	no**	yes	NC	yes	NC#	yes	*tissue samples **BMI, $no information on daytime of sample collection, # no info on sample randomization and QC samples
Vicente-Munoz 2015 urine	yes	no*	yes	yes	No**	yes	yes	yes	yes	NC#	*HW, **BMI, # no info on data transformation
Vouk 2016 PF	yes	no*	yes	NC$	yes	yes	yes	yes	NC#	NC##	*HW, $no information on daytime of sample acquisition, and freeze-thaw cycles, # no info on sample randomization, ## no info on data transformation and scaling
Ghazi 2016 serum	yes	no*	no**	NC$	no***	yes	NC	yes	yes	NC#	*HW, **rpm, ***no BMI, $no information on timing of sample processing, # no info on sample-to-sample normalization, data transformation and scaling
Vicente-Munoz 2016 plasma	yes	no*	yes	NC$	yes	yes	yes	yes	yes	NC#	*HW, $no information on daytime of sample acquisition and freeze-thaw cycles, # no info on sample-to-sample normalization, data transformation
Letsiou 2017 plasma	yes	NC	yes	NC$	NC*	no	yes	yes	no	NC#	Control patients with myoma, *fasting, $no information on daytime of sample acquisition, timing of sample processing, and freeze-thaw cycles, # no info on sample-to-sample randomization
Dominguez 2017 endometrial fluid	yes	no*	yes	NC$	yes	yes	yes	no**	yes	yes	*infertile patients excluded as controls, **not for controls, $no information on daytime of sample acquisition and freeze-thaw cycles
Chagovets 2017 tissue	yes	yes	yes	NC$	no*	yes	yes	yes	NC#	NC##	*wrong info about ethnicity, $no information on daytime of sample acquisition, # no info on sample randomization and QC samples, ## no info on data transformation and scaling
Dutta 2018 tissue, serum	yes	no*	no**	NC$	yes	yes	yes	NC	yes	NC#	*HW, **tissue and serum not described (reference), $no information on daytime of sample acquisition, timing of sample processing, and freeze-thaw cycles, # no info on data transformation and scaling
Li 2018 (FP) tissue	yes	yes	yes	NC$	yes	yes	no*	yes	yes	no	or*3 months after surgery, $no information on daytime of sample acqusition
Li 2018 (RBE) tissue	yes	yes	yes	NC$	yes	yes	yes	yes	NC#	no	$no information on daytime of sample acquisition, # no info on sample randomization

Braga 2019 plasma	yes	yes	yes	NC[$]	yes	yes	yes	NC	NC[#]	no	[$]no information on time between blood flow and centrifugation and freeze-thaw cycles, [#] no info on sample randomization
Starodubtseva 2019 plasma, PF	yes	no*	no**	NC[$]	no***	yes	yes	no	NC[#]	yes	*fertile patients undergoing myomectomy, ** collection of samples not described, *** no info about menstrual phase, [$]no information on daytime of sample acquisition and freeze-thaw cycles, [#] no info on sample randomization
Feider 2019	yes	yes	yes	NC[$]	no*	yes	yes	yes	yes	yes	*no data about BMI, menstrual phase, [$]no information on daytime of sample acquisition and storage time prior to metabolite extraction

Supplementary Table S3: QUADOMICS scoring of the included studies for cervical cancer.

Study/QUADOMICS	1	2	3A	3B	4	5	6	7	8	9	comments
**Cervical cancer (12)**											
Woo 2009 urine	yes	no*	yes	NC[$]	no**	yes	yes	no*	NC[#]	no	*HW, **no BMI, fasting status, [$]no information on daytime of sample acquisition, timing of sample processing, and freeze-thaw cycles, [#] no info on sample randomization and QC samples
Hasim 2012 plasma	yes	no*	no**	NC[$]	no***	yes	yes	no*	yes	no	*HW, **rpm, ***no BMI, menstrual phase, [$]no information on timing of sample processing, and freeze-thaw cycles
Hasim 2013 plasma	yes	nO*	no**	NC[$]	no***	no[#]	yes	no*	no[##]	no	*HW, **contradictory data about sample collection, *** no BMI, menstrual phase..., [$]no information on daytime of sample acquisition, timing of sample processing, and freeze-thaw cycles, [#] no sample preparation reported, [##] metabolomics not sufficiently described, no info on QC samples
Hou 2014 plasma	yes	yes	no*	NC[$]	no**	yes	yes	yes	NC[#]	NC[##]	*no info with regard to centrifugation, ** no BMI, [$]no information on daytime of sample acquisition and freeze-thaw cycles, [#] no info on sample randomization and QC samples, [##] no info on data transformation and scaling
Ye 2015 serum	yes	yes	no*	NC[$]	no**	yes	NC	NC	yes	no	*no information ontime before centrifugation, **no BMI, menstrual phase, [$]no information on timing of sample processing, and freeze-thaw cycles
Yin 2016 plasma	yes	no*	no**	NC[$]	no***	NC[#]	yes	NC	no	no	*myoma/CC?, **no data about tubes, centrifugation, *** BMI, [$]no information on daytime of sample acquisition, timing of sample processing, and freeze-thaw cycles, [#] no info on storage
Yang 2017 plasma	yes	no*	yes	NC[$]	no**	yes	yes	no*	yes	NC[#]	*HW, **no BMI, [$]no information on daytime of sample acquisition, timing of sample processing, and freeze-thaw cycles, [#] no info on sample-to-sample normalization, data transformation
Khan 2019 plasma	no*	no*	no**	NC[$]	no***	yes	NC	NC	NC[#]	no	HW *not for healthy, **rpm, ***no fasting, [$]no information on daytime of sample acquisition, timing of sample processing, and freeze-thaw cycles, [#] no info on sample randomization
Zhou 2019 plasma	yes	yes	no*	NC[$]	yes	yes	yes	yes	NC[#]	no	*rpm, [$]no information on daytime of sample acquisition, timing of sample processing, and freeze-thaw cycles, [#] no info on sample randomization

Ilhan 2019 lavage	yes	NC	yes	NC[$]	yes	yes	NC	NC	yes	no	HW HPV+, [$]no information on daytime of sample acquisition
Tokareva 2019 tissue	no*	NC**	no*	NC[$]	no*	NC[#]	yes	yes	NC[##]	no	*No data **CC and control, [$]no information on daytime of sample acquisition, timing of sample processing, and freeze-thaw cycles, [#] no info on storage, [##] no info on sample randomization and QC samples
Abudula 2020 tissue	yes	yes	yes	NC[$]	no*	no	yes	yes	no[#]	no	*no BMI,; [$]no information on daytime of sample acquisition, and freeze-thaw cycles, [#] metabolomics not sufficiently described

Supplementary Table S4: QUADOMICS scoring of the included studies for endometrial cancer.

Study/QUADOMICS	1	2	3A	3B	4	5	6	7	8	9	comments
**Endometrial cancer (14)**											
Ihata 2014 plasma	yes	NC*	no**	NC[$]	no***	no	NC	no	no	no	*BD and HW, **rpm, ***no menopausal status, BMI, [$]no information on daytime of sample aqcquisition, timing of sample processing, and freeze-thaw cycles
Trousil 2014 tissue	no*	no	no**	NC[$]	no***	yes	yes	yes	yes	NC[#]	Normal tissue*almost no data, **biopsy or sample after hysterectomy ***no clinical data, **** not written-clear for tissue samples?, [$]no information on daytime of sample acquisition and freeze-thaw cycles, [#] no info on data transformation and scaling
Jove 2016 tissue	no	no*	no	NC[$]	no**	no	yes	yes	NC[#]	no	*reproductive age women in control group ** no data about age, menopausal status, BMI..., [$]no information on daytime of sample acquisition and time between collection and storage, [#] no info onsample randomization and no QC samples used
Shao 2016 urine	yes	no	yes	NC[$]	no**	yes	yes	no	NC[#]	NC[##]	*BD and HW, **no clinical data, age, BMI, menopausal status, [$]no information on timing of sample processing, [#] no info on sample randomization, [##] no info on sample-to-sample normalization, data transformation, and scaling
Altadill 2017 tissue	yes	NC	yes	NC[$]	no*	yes	yes	yes	NC[#]	no	Bengin disease*age and BMI is missing, [$]no information on daytime of sample acquisition, [#] no info on sample randomization and type of QC sample
Audet-Delage 2018 (Front Pharm) serum	yes	NC	no*	yes	NC**	yes	yes	no***	no	no	Bengin disease *no data about collection and storage, **fasting status. *** HW
Audet-Delage 2018 (JSBMB) serum	yes	yes	no*	NC[$]	yes	yes	yes	yes	NC[#]	no	*no data about collection and storage, [$]no information on timing of sample processing, [#] no info on sample randomization
Troisi 2018 serum	yes	no	no**	yes	yes	yes	NC	no	NC[#]	NC[##]	*BD and HW, **no data about centrifugation, [#] no info on sample randomization, [##] no info on sample-to-sample normalization

Shi 2018 serum	yes	no*	no**	NC$	no***	yes	NC	no	NC#	NC##	*HW, **time before centrifugation, *** menopausal status, $no information on daytime of sample acquisition, timing of sample processing, and freeze-thaw cycles, # no info on sample randomization and QC samples, ## no info on sample-to-sample normalization, data transformation, scaling
Knific 2018 plasma	yes	NC	yes	NC$	no*	yes	yes	yes	NC#	yes	Benign diseases, *fasting status, $no information on daytime of sample acquisition, timing of sample processing, and freeze-thaw cycles, # no info on sample randomization
Bahado-Singh 2018 serum	yes	no*	no**	NC$	no***	yes	yes	no	NC#	NC##	*HW, **no data about serum collection, ***menopausal status, fasting?, $no information on daytime of sample acquisition, timing of sample processing, and freeze-thaw cycles, # no info on sample randomization and QC samples, ## no info on sample-to-sample normalization and scaling
Cummings 2019 tissue	no	NC	no*	NC$	no**	no#	yes	Yes	NC##	no	Normal and bengin tissue* no data about sample collection,** no clinical data, no age for CW, $no information on daytime of sample acquisition, # no storage temperature reported, ## no info on sample randomization and QC samples
Strand 2019 plasma	yes	yes	yes	NC$	no*	yes	yes	yes	no	no	*not fasting, $no information on daytime of sample acquisition, timing of sample processing, and freeze-thaw cycles,
Cheng 2019 CV fluid	yes	NC	no*	NC$	no**	yes#	NC	yes	yes	yes	Normal, benign diseases*not clear what was time between collection and storage, when in the menstrual, menopausal cycle has been collected, ** premenopausal and menopausal women, $no information on daytime of sample acquisition, timing of sample processing, and freeze-thaw cycles, # no sample storage reported

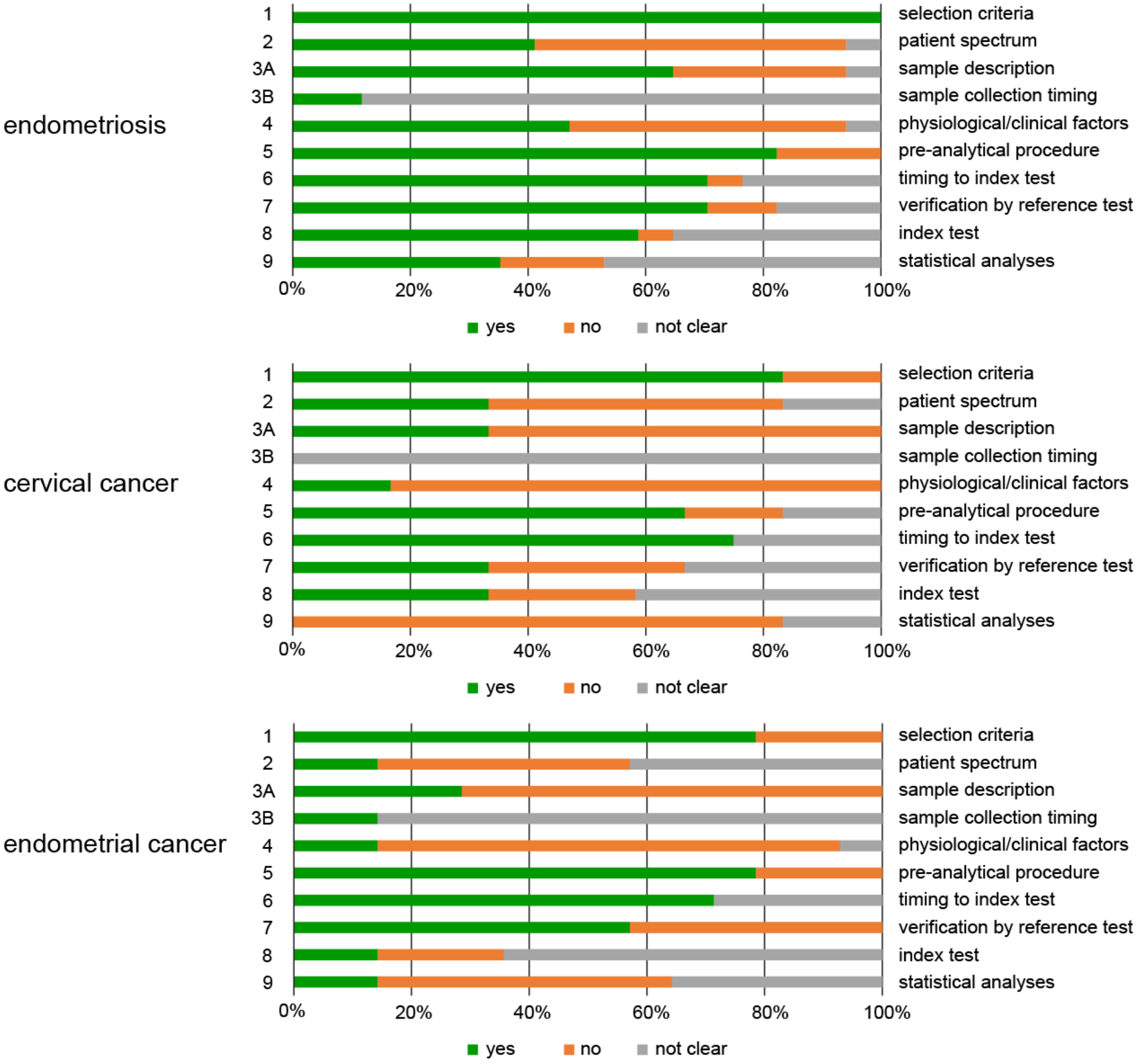

Supplementary Figure S1: QUADOMICS scoring of the included studies for endometriosis, cervical cancer, and endometrial cancer separately. Proportion of studies with answers "yes", "no", or "not clear" to each of the selected signaling questions. Each signaling question is numbered on the left, and a short description of each question is given on the right. The detailed scoring is given in Supplementary Tables S2, S3, and S4.

Supplementary Table S5: Metabolomics in uterine fibrosis.

Study/ Country	Extraction	Method	Sample	Control group	Case group	Findings
Heinonen *et al.* 2017 [1] Br. J. Cancer<br><br>Finland	not detailed	**Non-targeted**<br>RP/UPLC-MS/MS, HILIC/UPLC-MS/MS<br>Thermo Fisher Q-Exactive/Orbitrap	Tissue samples, stored at -80 °C	17 patients undergoing hysterectomy,<br>normal myometrial samples from the same patients	17 Patients with leiomyoma undergoing hysterectomy;<br>25 leiomyomas:<br>7 FH deficient, 7 mutation in *MED12*, 2 overexpression of *HMGA2*, 9 mutation negative	Leiomyomas/myometrium:<br>70 metabolites dysregulated,<br>↓ homocarnosine, haeme, biliverdin<br>**FH subtype**<br>↑ fumarate, N6-succinyladenosine, argininosuccinate, plasmalogens, diacylglycerols, and amino acids (Pro, Val, Leu, Ile); alteration in TCA cycle (malate, succinate, α-ketoglutarate, homocitrate) and pentose phosphate pathway<br>**MED12 subtype**:<br>↓ retinol, histamine, sphingolipids, and amino acids (Phe, Leu, Ile, Lys, Arg, Tyr, Trp).

Legend: FH, fumarase; MED12, mediator complex subunit 12; HMGA2, High-mobility group AT-hook 2

Supplementary Table S6: Metabolomics in endometriosis.

Study Country	Extraction	Method	Sample	Control group	Case group	Findings	Model
Vouk *et al.* 2012, Human Reprod. [2]<br><br>Slovenia	not detailed	**Targeted**<br>ESI-MS/MS<br>AbsoluteIDQ^{TM} p150 kit (Biocrates Life Sciences)<br>ABSciex API4000	**Plasma**, before laparoscopy, fasting samples, stored at -80 °C	52 healthy women undergoing sterilisation<br>(17 P, 11 LP/ES, 21 S, 2 ND, 1 MD),<br>Age: 40.6 ±3.1<br>BMI: 25.7 ±4.1	40 patients (14 OE, 20 OE + PE, 6 OE + PE + DIE)<br>(12 P, 8 LP/ES, 20 S)<br>Age: 33.3 ±6.1;<br>BMI: 20.9 ±2.7	↑ 8 metabolites: SMOH C16:1, SMOH C22:2, SM C16:1, PCae C32:2, PCae C34:2, PCae C36:1 PCae C34:0, PCae C30:0;<br>81 metabolite ratios	**SLR model**<br>SMOH C16:1 + PCaa C36:2/PCae C34:2 + age + BMI<br>SEN: 90%<br>SP: 84.3%<br>**AUC: 0.94**
Vicente-Muñoz *et al.*	plasma was mixed 1:1 with	**Non-targeted**<br>1 H-NMR	**Plasma** (overnight fasting, before	23 healthy women undergoing sterilization, (22 F, 1	50 patients with symptoms (OE and/or DIE	↑Val, fucose, choline-containing metabolites, Lys/Arg and lipoproteins	**PCA** revealed no significant difference,

2016, Fertil. Steril. [3]<br><br>Spain	75 mmol/L phosphate buffer pH 7.4, 5 mmol/L trimethylsilylpropionic acid-d4 sodium salt, 0.04% NaN_3 in D_2O	Bruker Avance III 600 MHz	surgery and anesthesia), stored at -80 °C	L), Age: 34.3 ±5.0, BMI: 22.0 ± 1.7<br>No MT or HT > 1 month before surgery	according to vaginal US) (6 I-II, 44 III-IV) confirmed by laparoscopy, (39 F, 11 L)<br>Age: 31.1 ± 5.5<br>BMI: 21.2 ± 1.6<br>No MT or HT > 1 month before surgery	↓ creatinine	OPLS-DA did not allow separation
Letsiou *et al*. 2017, Fertil. Steril. [4]<br><br>Belgium	not detailed	**Targeted**<br>UPLC-MS/MS (SteroIDQ kit), UPLC-ESI-Q-TOF<br>Agilent 6530, Waters TQMS, Waters Xevo	**Plasma** before anesthesia, stored at -80 °C	19 control patients (based on laparoscopy)<br>16 normal pelvis, 3 uterine myoma, (10 F, 9 L)<br>age: 41 ± 14, BMI: 26 ± 5, no HT.	25 patients (3 I, 6 II, 9 III, 7 IV), confirmed by laparoscopy, (18 F, 7 L)<br>Age: 32 ± 7, BMI: 24 ± 6, no HT.	↑ lauroylcarnitine, oleylcarnitine, myristoylcarnitine, tetradecenoylcarnitine, hexadecenoylcarnitine<br>↓ trimethylamine-N-oxide	**PLS-DA model**<br>long-chain acylcarnitines and trimethylamine-N-oxide<br>SEN: 81.8%<br>SP: 88.9%<br>PPV: 75%
Braga *et al*. 2019, Mol. Reprod. Dev. [5]<br><br>Brazil	methanol/chloroform (2:1, v/v)	**Non-targeted**<br>ESI-MS<br>Bruker Apollo II	**Plasma** in the morning of the day 3 of the menstrual cycle, fasted patients, stored at -20 °C	50 patients with male factor infertility, confirmed by laparoscopy, undergoing ICSI,<br>Age: 34.4 ± 2.5<br>BMI: 24.6 ± 3.1	50 infertile patients, confirmed by laparoscopy and histology undergoing intracytoplasmic sperm injection (III-IV),<br>Age: 33.6 ± 3.3<br>BMI: 24.5 ± 4.4	10 potential biomarkers (8 not identified, triacylglycerol, α-amino acid)	**PLS-DA model**<br>AUC: 0.90<br>SEN: 84%
Starodubtseva *et al*., 2019, Clin. Mass Spec. [6]<br><br>Russia	modified Folch method	**Non-targeted**<br>FIA-ESI-MS and FIA-ESI-MS/MS<br>Bruker Maxis Impact qTOF	**Plasma**<br>(prior anaesthesia, 12 h fasting)<br><br>Peritoneal fluid (during surgery)	20 fertile patients undergoing myomectomy,<br>Age: 33 ± 5<br>Caucasian: 95%<br>BMI: 24.1 ± 1.2	70 fertile endometriosis patients, confirmed by laparoscopy and histology<br>Age: 31 ± 6<br>Caucasian: 100%	Plasma:<br>↑ PE O-20:0, LPC 20:5, PC 36:5, PC 36:2, PC 38:6, PC 38:5, PC 40:9	**PLS-DA model**<br>including presumably all signals<br><br>Plasma:<br>SEN: 93%,<br>SP: 95%

			Snap frozen, stored at -80°C	4 infertility I; 2 infertility II; 3 miscarriage, 1 chronic pelvic pain syndrome;<br>no HT 6 month before surgery	BMI: 22.4 ± 1.1<br><br>35 I-II; 35 III-IV<br>49 infertility I, 21 infertility II; 14 miscarriage;<br>63 chronic pelvic pain syndrome<br>no HT 6 month before surgery	↓ LPC 16:0, DG 40:5, SM 34:1, PE O-34:1, PC 36:4, PC 38:7<br><br>PF:<br>↑ PE O-20:0, DG 38:2,<br><br>↓ LPC 16:0, DG 32:2, SM 34:1, PE O-34:1, PC 34:2, PC 34:1, PC 36:5, PC 36:6, PC 36:4, PC 36:3, PC 26:2, PC38:6, PC 38:4	PF:<br>SEN: 90%<br>SP: 95%
Dutta *et al*. 2012, Mol. BioSyst. [7]<br><br>India	serum was mixed 1:2 with D_2O containing 1 mM sodium salt of 3-(trimethylsilyl)propionic-2,2,3,3,d4 acid	**Non-targeted**<br>1 H-NMR Bruker Avance AV III 700 MHz	**Serum** stored at -80 °C	23 fertile women undergoing sterilisation, confirmed by laparoscopy, all S phase<br>Age <40; BMI <25<br>No HT > 3 months before surgery<br>age, BMI matched	22 patients (stages I-II), confirmed by laparoscopy, all S phase<br>Age <40; BMI <25<br>No HT > 3 months before surgery,<br>age, BMI matched	↑ lactate, 2-hydroxybutyrate, 3-hydroxybutyrate, Ala, glycerophosphatidylcholine, Val, Leu, Thr, Lys, succinic acid<br>↓ Glu, Ile, Arg<br>↑ anaerobic glycolysis, oxidative stress	**PLS-DA model**<br>SEN: 81.8%<br>SP: 91.3%<br>**AUC: 0.96**
Jana *et al*. 2013, BioMed Research International [8]<br><br>India	serum was mixed 1:2 with D_2O containing 1 mM sodium salt of 3-(trimethylsilyl)propionic-2,2,3,3,d4 acid	**Non-targeted**<br>1 H-NMR Bruker Avance AV III 700 MHz	**Serum** stored at -20 °C,<br>fasting samples	24 control women with tubal factor infertility, early F phase,<br>age: 24-40; BMI <25	26 endometrisis patients,confirmed by diagnostic laparoscopy, early F phase, age: 24-40; BMI <25	↑ lactate, 2-hydroxybutyrate, succinate, Lys, glcerophosphocholine, citric acid, pyruvate, adipic acid<br>↓ Ile, Leu, Arg, Asp, Ala, Glu, creatine<br>Altered metabolism of amino acids,<br>↑ glycolysis	**PLS-DA model**<br>SEN: 100%<br>SP: 91.6%<br>**AUC = 0.99**
Lee *et al*., 2014, J. Clin. Endocrinol. Metab. [9]	modified Bligh and Dyer extraction	**Targeted**<br>RP-LC-MS/MS Agilent 6460 Triple quadrupole	**Serum** samples, **peritoneal fluid** (PF),	24 subfertile patients, confirmed by laparoscopy, age 22-47, 9 P, 14 S,	38 subfertile patients with endometriosis, confirmed by laparoscopy, age:	↑total serum sphingomyelin, lactosyl-ceramide, ceramide, ceramide-1-phosphate, phosphatidylcholines<br>total PF phosphatidycholines	

Singapore			endometrial **tissue**, stored at -80 °C	no HT in the last 3 months before surgery,<br>PF: 26 subfertile patients, confirmed by laparoscopy, age 22-51, 10 P, 15 S	22-44, 11 I-II, 27 III-IV, 21 P, 17 S, no HT in the last 3 months before surgery. PF: 39 subfertile patients with endometriosis, confirmed by laparoscopy, age: 22-44, 13 I-II, 22 III-IV, 20 P, 18 S	↓ tissue total phosphatidylcholines<br><br>Serum: ↑ SM 18:1/20:0, SM 18:1/22:0, SM 18:1/22:1, GlcCer d18:1/24:1, SM 18:1/24:1, GlcCer d18:1/22:0, GlcCer d18:0/24:1, Cer d18:1/24:1, C1P d18:1/16:0, C1P d18:0/16:0, C1P d18:1/22:0,<br>Dysregulated sphingolipid metabolism	
Ghazi *et al*. 2016, Int. J. Reprod. BioMed. [10]<br><br>Iran	serum was mixed 10:1 with D_2O containing 3-trimethylsilyl-1-propanesulfonic acid sodium salt	**Non-targeted**<br>1 H-NMR Bruker 400 MHz	**Serum**<br>(fasting > 8h) stored at -80 °C	15 healthy women (diagnostic laparoscopy) without pelvic pain, pelvic inflammatory disease,<br>male factor infertility,<br>early F phase	31 infertile patients (stages II-III), confirmed by laparoscopy,<br>Age: 22-44<br>early F phase	↑ 2-OME1, 2-OME2, DHEA, androstenedione, aldosterone, deoxycorticosteron<br>↓ cholesterol,<br>7-dehydrocholesterol, taurocholic acid	**QDA model**<br>SEN: 76%<br>PPV: 71%<br>NPV: 78%
Vicente-Muñoz *et al*. 2015, Fertil. Steril. [11]<br><br>Spain	urine was mixed 10:1 with<br>1.5 mol/L potassium phsphate buffer<br>pH 7.4 containing 0.1% trimethylsilylpropionic acid-d4 sodium salt,	**Non-targeted**<br>1 H-NMR Bruker Avance III 500 MHz	**Urine**, first morning samples (overnight fasting), stored at -80 °C	36 healthy women undergoing sterilization (30 F, 6 L)<br>Age: 35.5 ± 5.2<br>No HT > 1 month before surgery	45 patients (6 I-II, 39 III-IV), confirmed by laparoscopy, (30 F, 15 L)<br>Age: 32.3 ± 6.6<br>No HT > 1 month before surgery	↑N-methyl-4-pyridone-5-carboxamide, guanidino-succinate, creatinine, taurine, Val, 2-hydroxyisovalerate, unknown metabolite U2<br>↓ Lys, unknown metabolites U1 and U6<br>↑ inflammation, oxidative stress	**PCA** revealed no significant difference

	and 0.05% NaN_3 in D_2O						
Vouk *et al*. 2016, J. Steroid Biochem. Mol. Biol. [12]<br><br>Slovenia	no extraction; sample used directly	**Targeted** ESI-MS/MS AbsoluteIDQ^{TM} p150 kit (Biocrates Life Sciences) ABSciex API4000	**PF**, collected at laparoscopy, stored at -80 °C	36 healthy women undergoing sterilization, 11 P, 9 LP/ES, 14 S, 2 ND, Age: 40.6 ± 3.2 years,<br>BMI: 16 normal, 14 overweight, 6 obese	29 OE patients, stages III and IV, confirmed by laparoscopy, 10 OE, 13 OE+PE, 6 OE+DIE+PE; 5P, 6 LP/ES, 18 S,<br>Age: 34.3 ± 6.3 years;<br>BMI: 4 underweight, 22 normal, 3 overweight	↓10 metabolites:<br>carnitine and acylcarnitines: C0, C8:1, C6C4:1, DC, C10:1; sphingomyelins: SM C16:1, SM C18:1;<br>phosphatidylcholines:<br>PCaa C38:3, PCaa C38:4, PCaa C40:4, PCaa C40:5	**SLR model**<br>(C0/PCae C36:0, PCaa C30:0/ PCae C32:2, age)<br>SEN: 82.8%<br>SP: 94.4%<br>**AUC: 0.94**
Dominguez *et al.* 2017, Biol. Reprod. [13]<br><br>Spain	methanol/chloroform (1:2, v/v)	**Non-targeted** UPLC-MS/MS	Samples of **endometrial fluid**, stored at -80 °C	13 control women no laparoscopy no histology,<br>mean age: 29 years,<br>BMI: 22.85<br><br>No HT > 3 month before<br>EF collected in the window of implantation<br><br>(LH surge + 7 days)	12 patients with OE, confirmed by laparoscopy and histology or positive ultra sound,<br>Mean age: 35 years,<br>BMI: 22.67<br><br>No HT > 3 month before<br>EF collected in the window of implantation	Difference in 123 /457 metabolites<br>95 ↓sphingolipids, glycerolipids;<br>PC 22:6/0:0,<br>28 ↑ mono or polyunsaturated TAG: TAG 46:0, TAG 48:0, TAG 48:1, TAG 50:4; CER d18:1/21:0, CER d18:1/23:0, PC O-42:6, SM d18:1/25:0, Cys, AC (6:0), AC (8:0), AC (10:0)<br><br>↓TAG with shorter acyl chains, less double bonds, phosphatidylethanolamines plasmalogens, CER, SM, monohexosylceramides<br>↑ TAG with longer acyl chains, higher number of double bonds	**SVM model**<br>123 metabolites<br>SP: 100%<br>SEN: 58.3%

Chagovets *et al.* 2017, Sci. Reports [14]<br><br>Russia	modified Folch extraction	**Non-targeted** HILIC-LC-MS (tissue spray ionization) Bruker Maxis Impact qTOF	Samples of eutopic and ectopic **endometrium**, frozen in liquid N2 and stored at -75 °C	30 patients with endometriosis, Age: 6 < 26, 7 26-30, 10, 30-36, 6, 36-41, 1> 41; 2 P, 27 LP/ES, 1 S, BMI: 2 < 18.5, 25, 18.5-25, 2, 25-30, 1 > 30; stage III-IV<br>No HT > 6 month before		↑ PC 38:4, PC 36:4, PC 38:5, PCO 38:5, PC 36:1, SM 34:1, SM 36:1, SM 42:3, PE O 36:5, PE 36:4, PE 36:1, PE O 38:5, PE O 40:5, PE 40:7<br>↓ PC 36:3, PC 38:3, SM 34:2, SM 42:2, PE 38:5	**OPLS-DA model** separate ovarian and peritoneal foci from eutopic endometrium
Dutta *et al*. 2018, Sci. Reports [15]<br><br>India	tissue was grinded with 6% per-chloric acid, neutralized and freeze-dried; resuspended in 100 mM sodium phosphate buffer<br>pH 7.4 in D_2O containing 1 mM sodium salt of 3-(trimethylsilyl)propionic-2,2,3,3,d4 acid	**Non-targeted** 1 H-NMR Bruker Avance III 700 MHz	Samples of eutopic **endometrium** and serum samples, before anestesia, stored at -80 °C	24 healthy patients undergoing sterilization, mid S phase,<br>age: 28.4 ± 3.2, BMI: 26.2 ±1.9, No HT in the last 3 month before surgery.	95 patients with endometriosis like symptoms (20 I, 13 II, 17 III, 45 IV), confirmed by laparoscopy and histology,<br>mid S phase, age: 29.4 ± 5.8, BMI: 26.0 ± 1.5, no HT in the last 3 months before surgery.	Tissue:<br>↓ Pro, Ala, Leu, Lys, Phe;<br>Serum samples patients (I and II):<br>Inverse association **tissue/serum**: Ala, Lys, Phe, Leu, positive association: Pro	**OPLS model**<br>for stage II<br>SEN:100%<br>SP: 83%
Li *et al*. 2018, Frontiers in Physiol. [16]<br><br>China	MTBE extraction	**Non-targeted** RP-UHPLC-ESI-HRMS ThermoScientific Q-Exactive	Samples of eutopic **endometrium**, stored in liquid N_2	20 infertile women without endometriosis, confirmed by laparoscopy, F phase,	21 patients, 14 I and 7 II stage, confirmed by laparoscopy, F phase, no HT in the last 3 months before surgery.	↓ PC (18:1/22:6), PC (20:1/14:1), PC (20:3/20:4), PS (20:3/23:1)<br>↑ PA (25:5/22:6)	**OPLS-DA model:** PC (18:1/22:6), PC (20:1/14:1), PC (20:3/20:4), PS (20:3/23:1), PA (25:5/22:6)<br>**AUC: 0.87**<br>SEN: 90.5%

				no HT in the last 3 months before surgery.<br>Age: 30.5 ± 3.0,<br>BMI: 21.2 ± 2.9	Age: 29.7 ± 3.1,<br>BMI: 20.8 ± 2.1		SP: 75.0%
Li *et al*. 2018, Reprod. Biol. Endocrinol. [17]<br><br>China	methanol extraction with bead-based homogenization	**Non-targeted** RP-/HILIC-UHPLC-ESI-HRMS ThermoScientific Q-Exactive	Samples of eutopic **endometrium**, stored in liquid N_2	37 infertile women without endometriosis confirmed by laparoscopy, F phase,<br>no HT in the last 3 months before surgery.<br>Age: 29.7 ± 3.4,<br>BMI: 21.9 ± 3.2	29 patients, 19 I and 10 II stage, 3 OE, confirmed by laparoscopy, F phase, no HT in the last 3 month before surgery.<br>Age: 29.7 ± 3.2,<br>BMI: 21.0 ± 2.1	↑ hypoxantine, Arg, Tyr, Leu, Lys, inosine, arachidonic acid, guanosine, xanthosine, lysophosphatidylethanolamine, Arg<br>↓ uric acid<br>↑purine metabolism	**LR model:**<br>uric acid, hypoxanthine and lysophosphatidylethanolamine<br>**AUC: 0.87**<br>SEN: 66.7%<br>SP: 90.0%
Feider *et al*. 2019, Sci. Reports [18]<br><br>USA	sectioned with CryoStar NX50 cryostat	**Non-targeted** DESI-MS ThermoScientific LTQ-Orbitrap Elite	Eutopic and ectopic **endometrial tissue**, stored at -80°C	22 endometriosis patients provided eutopic tissue,<br>Age: 19-54 years, no exclusion criteria	76 endometriosis patients provided ectopic tissue from peritoneum, rectum ligaments, ovaries fallopian tubes<br>Age: 19-54 years, no exclusion criteria	↑ hexose, FA 18:2, FA 18:1, PS 18:1/18:0<br>↓ Iodine, lactate, FA 16:0, FA 20:4, FA 20:3, FA 22:4, PI 18:0/20:4, PI 18:0/ 20:3	**LS method**<br>Training set (59)<br>Validation set (14)<br>Independent set (25)

Legend: AC, acylcarnitines; Cer, ceramide; C1P, ceramide-1-phosphate, DG, diacylglycerol; DHEA, dehydroepiandrosterone; DIE, deep infiltrating endometriosis; EF, endometrial fluid; ESI-MS/MS, electrospray ionisation tandem mass spectrometry; FA, fatty acid; GlcCer, glucosylceramide; HT, hormone therapy; ICSI, intracytoplasmic sperm injection; L, luteal; LP/ES, late proliferative/early secretory phase; LR, logistic regression; LS, lassso statistical; LPC, lysophosphatidylcholine; MD, missing data; NA, not applicable; ND, not determined; OE, ovarian endometriosis; OPLS, orthogonal partial least squares; PCA, principal component analysis; PE, peritoneal endometriosis; P, proliferative phase; PA, phosphatidic acid; PCae; PCaa, glycerophospholipids, PF, peritoneal fluid; PLS-DA, Partial least squares discriminant analysis; PPV, positive predictive value; PS, phosphatidylserine; QDA, quadratic discriminant analysis; S, secretory phase; SEN, sensitivity; SM, sphingomyelin; SLR, stepwise logistic regression; SMOH, hydroxysphingomyelin; SP, specificity; SVM, support vector machine; TAG, triacylglycerol, I-V stage of endometriosis.

Supplementary Table S7: Metabolomics in cervical cancer.

Study Country	Extraction	Method	Sample	Control group	Case group	Findings/ Models
Hasim *et al.* 2012, Exp. Therapeutic Med. [19]<br><br>China	plasma was mixed 1:2 with 0.9% NaCl and 20% D_2O in 80% H_2O	**Non-targeted** 1H NMR Varian Innova 600	**Plasma** samples after overnight fasting, prior to treatment or at routine check up, stored at -80°C	38 healthy controls, age: 41.6 ±0.3 years	38 patients with CIN (2 CIN I, 31 CIN II, 5 CIN III) 39.6 ± 0.7 years,<br>38 patients with CSCC; (18 IIb, 16 IIIb, 4 IVb)<br>45.6 ±0.3 years	**OPLS-DA**<br>CIN versus HC: SEN = 91.6%,<br>CSSC versus HC: SEN = 100%<br>22 metabolites separate CSCC, CIN, and HC:<br>CIN versus HC:<br>↑ VLDL, acetone, unsaturated lipids and carnitine<br>↓ creatine, lactate, Ileu, Val, Ala, Gln, His, Gly, acetylcysteine, myo-inositol, choline, glycoproteins<br>CSCC versus HC:<br>↑ acetate, formate<br>↓ creatine, lactate, Ileu, Leu, Val, Ala, Gln, His, Tyr<br>CSCC versus CIN<br>difference in acetone, acetate, formate, glycoprotein, α-glucose and β-glucose
Hasim *et al.* Mol. Biol. Rep. 2013 [20]<br><br>China	protein precipiation with acetonitrile	**Targeted** RP-HPLC	**Plasma** samples, after overnight fasting,<br>stored at -80 °C	35 healthy controls, (age matched to CSCC)	22 CSCC patients (8 FIGO IIa, 14 FIGO IIIb, 10 G1, 4 G2, 8 G3, 8 LNM) age: 52.7 (42-67)<br>26 CIN patients 8 10 CIN II, 16 CIN III) age: 46.3 (29-56)	CIN and CSSC versus HC:<br>↓ Asp, Gln, Asn, Ser, Gly, His, Tyr, Val, Met, Lys, Ileu, Leu, Phe and taurine (gradually reduced from CIN to CSCC)<br>CIN versus HC:<br>↑ Arg, Thr<br>CSCC versus HC:<br>↓ Arg, Thr<br>**PLS-DA model**
Hou *et al.* Mol. BioSyst. 2014 [21]<br><br>China	methanol extraction	**Non-targeted** RP-UPLC-ESI-MS Waters Micromass QTOF	**Plasma** samples, fasting patients, stored at -80 °C	Patients with CC after neoadjuvant chemotherapy (three cycles of paclitaxel and carboplatin);<br>15 patients with complete response (CR), Age: 50.7 ± 11.0; 7 pre-, 8- postmenopausal, 1 IB2, 8 IIA, 6 IIB, 15 LVI-, 3 G1, 8 G2, 4 G3<br>14 partial response (PR), Age: 50.0 ± 7.5; 4 pre-, 10-postmenopausal, 1 IB2, 4 IIA, 9 IIB, 11 LVI- 3 LVI+, 2 G1, 8 G2, 4 G3		**PLS-DA**<br>562 peaks identified;<br>Metabolites selected based on VIP > 1, p <0.05<br>CR:PR:SD: ↓L-Val, L-Trp, DHEA-S<br>PR:SD: ↓Cer (d18:0/12:0)<br>CR:PR: ↑ Cer (d18:0/12:0)

				9 patients with stable disease (SD, Age: 46.4 ± 9.8; 1 pre-, 8- postmenopausal, 2 IB2, 4 IIA, 3 IIB, 9 LVI-, 1 G1, 4 G2, 4 G3		**Predictive models:**<br>L-Val<br>CR:SD **AUC = 0.73**<br>CR+PR:SD **AUC = 0.72**<br>L-Trp<br>CR:SD **AUC = 0.92**<br>CR+PR:SD **AUC = 0.82**<br>L-Val + L-Trp<br>CR:SD<br>SEN: 87%<br>SP: 80%<br>**AUC= 0.94**
Yin *et al.* 2016, Tumor Biol. [22]<br><br>China	methanol extraction	**Non-targeted**<br>RP-UPLC-ESI-MS<br>Waters Micromass QTOF	**Plasma,**<br>12 h fasting patient,	93 patients with uterine fibroids<br><br>Training: 47 controls<br>Age: 45.2 ± 7.8, 39 pre-menopause, 8 postmenopause<br><br>Validation: 45 controls<br>Age: 47.6 ± 7.1, 34 pre-menopause, 12 postmenopause	89 SCC patients<br><br>Training: 45 SCC, 8 I, 37 II,<br>Age: 47.6 ± 9.0, 22 pre-menopause, 23 postmenopause<br><br>Validation: 44 SCC, 8 I, 36 II,<br>Age: 46.4 ± 9.6, 17 pre-menopause, 27 postmenopause	**PLS-DA**<br>metabolites selected based on VIP < 1, AUC < 0.75:<br>(IDENTIFIED BASED ON MASS ONLY)<br>↓ PC (18:2/20:5), PC (18:1, 15:0)<br>↑ LysoPC (18:0), LysoPC (10:0)<br>Validation:<br>Combination of 4 metabolites<br>**AUC: 0.97**<br>SEN: 93.2%<br>SP: 91.3%<br><br>Validation by ELISA<br>↓ total PC<br>↑ total LysoPC
Yang *et al.* 2017 Scientific Reports [23]<br><br>China	protein precipiation with acetonitrile	**Non-targeted**<br>RP-UPLC-Q-TOF-MS<br>MS/MS identification<br>Agilent 6520 Q-TOF MS	**Plasma**, stored at -80 °C<br>fasting patients	149 control healthy women<br>Training: 80 controls<br>Age: 49.8 (41.0-69.0)<br>Test set: 69 controls<br>Age: 54 (41.0-68.0)	136 patients with CC<br>47 stage I, 64 stage II, 1 stage I, 24 NA<br>Training: 70 CC<br>Age: 32.8-66.7<br>Test set: 66 CC<br>Age: 49.8 (40.9-66.1)	Metabolites selected based on p < 0.05 and VIP > 1:<br>34 in ESI+ mode, 28 in ESI- mode<br>CC patiens ↓ 55 ↑ 7 metabolites<br>5 metabolites selected:<br><br>Bilirubin, LysoPC (17:0), n-oleoyl Thr, 12-hydroxydodecanoic acid, tetracosadexaenoic acid<br>**AUC: 0.99**

						SEN: 98%<br>SP: 99%	
Khan *et al.* 2019<br>Cancers [24]<br><br>Korea	chloroform: methanol (2:1, v/v) extraction	**Non-targeted**<br>RP-UPLC-QTOF-MS<br>(52 in positive and 40 in negative mode)<br>ABSciex Triple TOF 5600<br>**Targeted**:<br>RP-UPLC-TQ-MS<br>Agilent 6495 Triple Quadrupole MS	**Plasma**, stored at -80 °C	Non-targeted:<br>137 HW<br>Targeted:<br>69 HW<br>Age: 48 (43-51)<br>BMI: 21.6 (20.5-23.2)<br>HPV+ 30<br>postmenopausal: 28<br>smoking: 8	Non-targeted:<br>108 CIN 1<br>54 CIN 2/3<br>108 CC<br>Targeted:<br>55 CIN 1<br>Age: 35 (31-40)<br>BMI: 20.6 (19.4-21.9)<br>HPV+ 30<br>Postmenopausal: 4<br>smoking 18<br>42 CIN 2/3<br>Age: 39.5 (33-49)<br>BMI: 20.8 (19.4-23.4)<br>HPV+ 30<br>Postmenopausal 8<br>smoking 7<br>60 CC<br>Age: 50 (42-51)<br>BMI: 23.2 (20.6-25.7)<br>HPV+ 47<br>Postmenopausal 34<br>Smoking 7	Non-targeted:<br>**PCA two clusters:**<br>healthy+ CIN 1 versus CIN 2/3 and CC<br>FDR impact value > 0.3 and $p < 0.05$<br>N/CC, CIN 1/CC, N+ CIN 1/CIN 2/3 +CC<br>Ala, Asp, Glu, Arg and Pro metabolism, taurine and hypotaurine and pyruvate metabolism<br>28 metabolites significantly changed; top (based on AUC and hierarchical cluster analysis) 7: AMP, Asp, Glu, hypoxanthine, lactate, Pro, pyroglutamate<br><br>Targeted (validation):<br>↑AMP, Asp, Glu, Hypoxanthine, lactate, Pro, pyroglutamate<br>**Model**<br>N/CIN 2/3,<br>**AUC = 0.82**<br>N/ CC<br>**AUC = 0.83**<br>CIN 1/ CIN 2/3<br>**AUC = 0.72**<br>CIN 1/ CC<br>**AUC = 0.78**<br>N+CIN/ CIN 2/3/CC<br>**AUC = 0.78**	
Zhou *et al.* 2019<br>Medicine [25]<br><br>China	protein precipitation with methanol:acetonitrile (1:1, v/v)	**Non-targeted**<br>RP-UPLC-Q-TOF-MS	**Plasma**, stored at -80 °C<br>12 h fasting patients	30 CC patients before treatment,18 Figo II, 12 III<br>Age: 52.2 ± 8.0, BMI: 24.9 ± 4.1, 14 postmenopausal<br><br>30 CC patients with poor prognosis (local recurrence, distant metastases, blood, imaging), 5 Figo I, 16 II, 9 III, 11 first treatment surgery, 19 chemotherapy<br>Age: 53.3 ± 8.6, BMI: 23.9 ± 2.5, 13 postmenopausal		VIP > 1, $p < 0.05$<br>**CC before/poor prognosis:**<br>258 differential metabolites<br>**CC before/ good prognosis:**<br>228 metabolites	**Models**<br>Phthalic acid, D-maltose, PG (12:0/13:0), LacCer (d18:1/16:0), PC (15:0/16:09)<br>**CC before/poor prognosis:**

				30 CC patients with good prognosis (without local recurrence, blood, imaging test, 4 Figo I, 21 II, 5 III, 9 first treatment surgery, 21 chemotherapy<br>Age: 52.4 ± 8.0, BMI: 25.1 ± 2.8, 17 postmenopausal<br><br>122 patients with benign gynecological diseases (BGD), *54 leiomyoma, 7 adenomyosis, 18 endometrial cyst, 14 cystic teratoma, 13 mucinous cyst, 1 serous cyst adenoma, 4 fibroma, 9 simple cyst, 2 others;* Age: 45 (23-82) years<br><br>240 healthy women (HW); Age: 58 (32-82) years<br>Training set: 120 HW<br>Validation set: 120 HW and 122 BGD		**Good/poor prognosis:**<br>174 metabolites<br><br>31 common metabolites: Gycerophospholipids (PE, PC, PG, PS), sphingomyelins, glycosphingolipids, Lyso PC, phthalic acid,...	**AUC: 0.97**<br>SEN: 94%<br>SP: 87%<br><br>**CC before/ good prognosis:**<br>**AUC: 0.97**<br>SEN: 92%<br>SP: 89%<br><br>**Good/poor prognosis:**<br>**AUC: 0.91**<br>SEN: 86%<br>SP: 80%
Ye *et al*. 2015 Eur. J. Gynaecol. Oncol. [26]<br><br>China	plasma was mixed 1:2 with D_2O	**Non-targeted**<br>1H NMR Varian Unity Inova 600	**Serum** samples, fasting patients, stored at -60 °C	22 Chronic cervicitis<br>Age: 31 (22-43)<br>9 CIN<br>Age: 33 (24-43)	18 CC<br>Age: 40 (35-46)	**PLS-DA**<br>20 metabolites differ between CC/CIN and cervicitis<br>12 metabolites with statistically significant difference:<br>↓ formate, Tyr, β-glucose, inositol, carnitine, Gln, Val, Ile, ↑ Gly, Ala, VLDL<br>CC versus CIN ↓ acetate, CC versus cervicitis ↑ acetate	
Woo *et al*. 2009 Clin Chem Acta [27]<br><br>Korea	solid-phase extraction; diethylether extraction; followed by derivatizatio n	**Targeted**<br>GC-MS + RP-LC-MS; only steroids and nucleosides); **non-targeted** (GC-MS) Thermo Finnigan Trace 2000 GC Agilent 5890A	**Urine** samples, collected before the surgery, stored at -20°C	22 controls,<br>age 45.1 ± 9.76 years, no pathological evidences of breast,<br>cervical, and ovarian cancers;<br>pre-menopausal: n=8, age 45.1 ± 6.73 years	12 patients with CC (n=12, age 46.7 ± 19.2 years)<br>pre-menopausal: n=7, age 36.9 ± 14.2 years	**PLS-DA** discriminated pre-menopausal CC and OC cases from controls (targeted and non-targeted separately);<br>CC and OC versus HW<br>↑ 4-androstene-3,17-dione,<br>1-methyladenosine, 3-methyluridine no biomarker identified for CC	

Ilhan *et al*. 2019, EbioMedicine [28]<br><br>USA	protein precipitation with ethanol	**Non-targeted** RP/HILIC-UPLC-MS/MS ThermoScientific Q-Exactive	**Cervicovaginal lavage**, stored at -80 °C	pre-menopausal HW<br>18 HPV-, Age: 40.4 ± 7.0, BMI: 31.4 ± 11.5<br><br>11 HPV+, Age: 36.4 ± 9.5, BMI: 31.6 ± 6.6<br><br>no significant difference in age and BMI	12 patients with low-grade squamous intraepithelial lesions (LSIL), Age: 35.1 ± 7.3,<br>BMI: 27.4 ± 4.6<br><br>27 high-grade squamous intraepithelial lesions (HSIL), Age: 38.3 ± 8.5, BMI: 30.7 ± 7.6<br><br>10 invasive cervical carcinoma (ICC), Age: 38.9 ± 9.1,<br>BMI: 27.1 ± 7.0	Metabolites discriminate<br>HW (HPV+/HPV-): N-acetyltaurine (AUC = 0.88), deoxycarnitine, C-glycosyltryptophane<br>HW (HPV-)/ LSIL: pentose acid (AUC = 0.83), tartrate, 1-methylhypoxnthine<br>HW (HPV-)/ HSIL phosphoetanolamine (AUC = 0.84)<br>ICC/ HW (HPV-): 3-hydroxybutyrate (AUC = 0.92), eicosenoate, oleate/vaccenate, salicylate
Tokareva *et al*. 2019, J. Mass Spectrom. [29]<br><br>Russia	modified Folch extraction	FIA-ESI-MS/MS Bruker Maxis Impact qTOF	**Tissue** samples	10 border tissue	10 CC tissue	**OPLS-DA**<br>438 peaks (m/z 600-900), 152 with significant difference (38 lipids)<br>**Models**<br>Non-polar glycerolipids AUC = 0.95<br>Phosphatidylethanolamines AUC = 0.86
Abudula *et al.* 2020, Bosn. J. Basic Med. Sci. [30]<br><br>China	not detailed	**Non-targeted** 1H NMR Varian Unity Inova600	**Cervical tissue** stored at -80 °C	11 control patients (1 HPV+)<br>Matched by age and childbirth	21 SCC (21 HPV+)<br>20 CIN II-III (20 HPV+), Age: 45.2 (25-69)	good NMR spectra for 32 samples out of 52: 16 SCC and 17 CIM all HPV+ versus 10 NC HPV-<br>17 metabolited differentiate between two groups<br>**OPLS-DA**<br>separates SSC/NC, CIN/NC, SCC/CIN<br>SCC/CIN and NC:<br>↑ LDL, lactate, Ala, ↓α/β Glu, Typ, Phe<br>SCC/NC:<br>↓ Ile, methylproline, creatine, acetate, inositol

Legend: CC, cervical cancer; CIN, cervical intraepithelial neoplasia; CSSC, cervical squamous cell carcinoma; CER, ceramides; HPV, human papiloma virus; ESI-MS/MS, electrospray ionisation tandem mass spectrometry; G, grade; HRT, hormone replacement therapy; LNM; LVSI, lymphovascular space invasion; MeO, methoxy; MD, missing data; MI, myometrial invasion; NA, not available; ND, not determined; OPLS-DA, orthogonal partial least squares

discriminant analysis; PCA, principal component analysis; PCae; PCaa, glycerophospholipids; PC, phosphatidylcholine; PE, phosphatidylethanolamine; PG, phosphatidylglycerol; PLS-DA, Partial Least Squares Discriminant Analysis; S, secretory phase; SEN, sensitivity; SM, sphingomyelin; SMOH, hydroxysphingomyelin; SCC, squamous cervical cancer; SP, specificity; VIP, variable importance in projection; QTOF, quadrupole time of flight

Supplementary Table S8: Metabolomics in endometrial cancer.

**Study Country**	**Extraction**	**Method**	**Sample**	**Control group**	**Case group**	**Findings/ Model**	
Ihata *et al.* 2014, Int. J. Clin. Oncol. [31]<br><br>Japan	not detailed	**Targeted**<br>HPLC-ESI-MS	**Plasma,** stored at -80 °C, overnight fasting	122 patients with benign gynecological diseases (BGD), *54 leiomyoma, 7 adenomyosis, 18 endometrial cyst, 14 cystic teratoma, 13 mucinous cyst, 1 serous cyst adenoma, 4 fibroma, 9 simple cyst, 2 others;* Age: 45 (23-82) years<br>240 healthy women (HW); Age: 58 (32-82) years<br><u>Training set:</u> 120 HW<br><u>Validation set</u>: 120 HW and 122 BGD<br>Age and BMI matched	80 EC; 48 I, 9 II, 15 III, 8 IV; 40 G1, 15 G2, 6 G3, 19NA; 54 endometrioid, 6 adenosquamous, 6 serous, 3 clear cell, 1 mucinous, 8 carcinosarcoma, 1 squamous, 1 poorly differentiated; Age: 58 (32-80) years;<br><u>Training set:</u> 40 EC patients<br><u>Validation set:</u> 40 EC patients	Training set:<br>↓ His, Trp, Val, Phe, Asp, Ser, Leu and Met<br>↑ ornithine, Ile, Pro<br><br>**LR models:**<br><u>EC/HW</u><br>His, Ile, Val and Pro:<br>**AUC= 0.94**; SEN= 60%, SP= 98.3%<br>CA-125: AUC= 0.80	<u>EC/BGD</u><br>His, Ile, Val, and Pro:<br>**AUC= 0.83**<br>CA-125: AUC= 0.60<br><br><u>EC I /HW</u><br>His, Ile, Val, and Pro:<br>**AUC= 0.91**<br>CA-125: AUC= 0.79<br><br><u>EC II-IV /HW</u><br>His, Ile, Val, and Pro:<br>**AUC= 0.99**<br>CA-125: AUC= 0.83
Knific *et al.* 2018<br>J. Steroid Biochem. Mol. biol. [32]<br><br>Slovenia	no extraction; sample used directly	**Targeted**<br>FIA-ESI-MS/MS<br>Absolute/DQ™ p150 kit (Biocrates Life Sciences)<br>ABSciex API4000	**Plasma** samples, collected and processed according to SOP, stored at -80 °C	65 patients with prolapsed uterus or myoma, Age: 63.2 ± 9.4	61 EC patients, 9 with LVI, 16 with > ½ MI<br>Age: 65.1 ± 8.7, no difference between groups in age, menopausal status, medication intake, diabetes, hypertension, smoking status	↓ 3 metabolites: PCaa C40:1, PCaa C42:5, PCaa C42:6, 166 metabolite ratios<br>↑ total short-chain and long chain acylcarnitines, Pro/Tyr<br>**LR model**<br>**EC/controls**<br>C16/PCae C40:1, Pro/Tyr, PCaa C42:0/PCae C44:5<br>**AUC: 0.84**<br>SEN: 85.3%<br>SP: 69.2%<br>**Detection of MI:**	

						SMOH C14:1/SMOH C24:1, PCaa C40:2/PCaa C42:6<br>**AUC: 0.86**<br>SEN: 81.3%<br>SP: 86.4%<br>SMOH C14:1/SMOH C24:1, C16:2/lyso PCa C16:1<br>**AUC: 0.85**<br>SEN: 75%<br>SP: 72.7%<br>SMOH C14:1/SMOH C24:1, PCaa C40:2/PCae C40:1<br>**AUC: 0.85**<br>SEN: 68.8%<br>SP: 97.7%<br>SMOH C16:1/SMOH C24:1, PCaa C34:4/PCae C34:3<br>**AUC: 0.85**<br>SEN: 81.2%<br>SP: 77.3%<br><br>**Detection of LVI:**<br>PCaa C34:4/PCae C38:3, C16:2/PCaa C38:1<br>**AUC: 0.94**<br>SEN: 88.9%<br>SP: 84.3%
Strand *et al.* 2019, Metabolites [33]<br><br>Norway	no extraction; sample used directly	**Targeted**<br>LC-MS/MS Absolute/IDQ™ p180 kit (Biocrates Life Sciences)<br>ABSciex QTrap4000	**Plasma** samples, stored at -80 °C	EC patients with long and short survival:<br>20 EC patients with short survival, Age: 75 (63.6-81.5), 13 MI, 8 endometrioid, 5 serous, 5 carcinosarcoma, 2 non-endometrioid, 3 G1, 2 G2, 3 G3, 18 stage I, 2 stage II<br>20 EC patients with long survival, Age 67 (56.0 -77.0), 6 MI, 7 endometrioid, 3 clear cell, 3 serous, 6 carcinosarcoma, 1 non-endometrioid, 3 G1, 2 G2, 2 G3, 18 stage I, 2 stage II<br>Patients were matched for FIGO stage, histology, grade, age, and BMI		**Long/short survival:**<br>↓ methionine sulfoxide (MetSO), hydroxypropionylcarnitine (C3-OH)<br>**Model 1:** MetSO, serotonin, spermine, C3-OH, PCaa C36:5, SM C20:2<br>**AUC = 0.82**<br>**Model 2:** MetSO, serotonin, spermine, C3-OH, PCaa C36:5, SM C20:2, spermidine, butenylcarnitine (C4:1), lyso PCaa C18:2 and lysoPCaa C24:0<br>**AUC = 0.935**

						**Model 3:** MetSO, serotonin, spermine, C3-OH, PCaa C36:5, SM C20:2, spermidine, C4:1, lyso PCaa C18:2, lysoPCaa C24:0, Asp, dimethylarginin, hexose, PC ae C30:1<br>**AUC = 0.965**
Audet-Delage *et al.* 2018 J Steroid Biochem Mol. Biol. [34]<br><br>Canada	ethyl acetate:chlorobutane (25:75, v/v) followed by derivatization with dansyl chloride	**Targeted** GC-MS (13 unconjugated steroids), RP-LC-MS/MS (14 conjugated steroids, catechol estrogens) ABSciex API5500 QTrap	**Serum** samples collected before surgery and one month after surgery, stored at -80 °C	110 healthy postmenopausal women,<br>Age: 58.3 ± 5.6<br>OC: 145 no, 91 yes, 10 missing<br>HRT: 157 never, 80 ever, 9 missing	246 EC cases, 202 type I, 44 type II, 90 G1, 94 G2, 61 G3, 1 NA, 197 stage I, 12 II, 28 III, 9 IV, 187 < 50% MI, 59 > 50 % MI, 183 NO LVI, 58 LVI, 220 no relapse, 26 relapse (follow up 65.5 months) 5 year recurrence 24 cases,<br>Age: 65.1 ± 8.9<br>OC: 19 no, 91 yes;<br>HRT: 40 never, 70 ever	**BMI:** ↑E3, E1-S, E1, E2, 2MeO-E1<br>**MI** ↓ E3<br>**Recurrence:**<br>↑ E1-S<br>↓ E3<br>**EC (after)/ EC (before):** ↓ all steroids except ↑4MeO-E2<br>EC (after) ≈ HW¸↑4MeO-E2<br>**EC (type 1 and type 2 before) /HW:**<br>↑DHEA, 5-diol,4-dione,testosterone, DHT, ADT-G, 3a-Diol_G, 3a-Diol-17G, E1-S, E1, E2<br>**EC (type 2, before) / HW:** ↑ DHEA, 5-diol, 4-dione, testosterone, ADT-G
Audet-Delage *et al.* 2018 Frontiers in Pharmacology [35]<br><br>Canada	protein precipitation with methanol;<br><br>heptane/ethyl acetate/butanol/methanol extraction	**Non-targeted** Metabolon platform RP-UPLC-MS/MS ThermoFisher Q-Exactive; Sciex SelexIon-5500QTrap	**Serum**, fasting patients, stored at -80 °C	18 control women (benign conditions) postmenopausal, no HRT for the last 3 weeks<br>Age: 58.9 ± 10.4, BMI 27.5 ± 7.2	26 EC, 24 type 1, 12 type 2, non-recurrent (NR), recurrent (R) postmenopausal, no HRT for the last 3 weeks<br>NR: Age: 66.3 ± 8.3, BMI 28.4 ± 7.0<br><br>R: 12 endometrioid, 6 serous<br>Age: 67.5 ± 9.4, BMI 28.0 ± 6.4	1592 metabolites analyzed,<br>**EC/C:** 137 metabolites, ↑115 (acylcholines, monoacylgycerols, acylcarnitines), ↓22 (free fatty acids)<br>Peptides and aminoacids: spermine and isovalerate, glycylvaline , gamma-glutamyl-2-aminobutyrate **AUC = 0.92**<br>**Type I/type II:** 98 metabolites, ↑ 30 (bradykinin, sulfated androgens)<br>↓ 68 (heme, saturated long-chain acylcarnitine, choline, sarcosine, Gly)<br><br>**R/ NR:** 104 metabolites (80 involved in lipid metabolism)<br>↑ monoacylglycerols, docosahexaenoyl carnitine, 2-hydroxypalmitate, 2-hydroxystearate ↓ Ser, Thr<br><br>**R/ NR**: 2-oleoylgycerol and TAG 42:2-FA12:0 ,

						**AUC = 0.90**<br><br>**Type 1 R cases** ↓ bile acids (taurodeoxycholate, glycodeoxycholate and taurocholate) ↑ phosphorylated fibrinogen cleavage peptide<br>**Type 2 R cases** ↑ sphingolipids (ceramides, dihydroceramides, lactosylceramides)
Troisi *et al.* 2018<br>J Proteome Research [36]<br><br>Italia	extraction with MetaboPrep GC kit (Theoreo)	**Non-targeted**<br>GC-MS Shimadzu GC-2010 Plus	**Serum** samples, fasting samples, stored at -80 °C	1st group:<br>80 HW<br>Age: 60 (55-65), BMI 27.8 (24.2-29.0)<br><br><br>2nd group:<br>50 HW<br>Age: 65 (59-69), BMI 27.1 (23.9-30.5)	1st group:<br>88 EC patients, 67 type I, 21 type II, 2 G1, 53 G2, 33 G3, 36 stage I, 45 II, 7 III<br>Age: 68 (62-68)<br>BMI 28.3 (25.1-30.3)<br><br>2nd group:<br>30 EC, 23 type I, 7 type II, 4 G1, 22 G2, 4 G3, 12 stage I, 15 II, 3 III Age: 66 (61-72), BMI 28.9 (26.3-31.1); 30 ovarian cancer, Age: 65 (59-69), BMI 27.1 (23.3-29.7); 10 benign diseases (hyperplasia, polyps, bleeding) Age: 63 (57-66), 27.8 (24.8-32.1)	259 metabolites determined consistently<br>**PLS-DA models** (also LDA, NB, DT, RF, K-NN, ANN, SVM)<br>**EC/HW:**<br>↑ lactic acid, homocysteine,3-hydroxybuthyrate<br>↓ linoleic acid, stearic acid, myristic acid, Thr, Val, progesterone<br>Accuracy: 0.99<br>SEN: 97%<br>SP: 98%<br><br>**type 1/ type 2:**<br>↓ lactic acid, cystine, Ser, malate, Glu, homocysteine<br>↑ progesterone<br>Accuracy: 0.93<br>SEN: 96%<br>SP: 86%
Shi *et al.* 2018 Cancer Science [37]<br><br>China	protein precipitation with methanol	**Non-targeted**<br>RP-UPLC-ESI-Q-TOF/MS Waters MicromassQ/TOF	**Serum** from fasting patients, stored at -80 °C	46 HW<br>Age: 57 ± 10, BMI 25.8 ± 3.1	46 EC patients type 1, 27 stage Ia, 19 IIb, 20 G1, 13 G2, 13 G3<br>Age: 52 ± 8, BMI 26.9 ± 5.1	**PLS-DA and OPLS-DA model:**<br>7646 in positive mode, 2579 negative mode<br>↑ Phe, indoleacrylic acid, phoshocholine (PC), lyso-platelet-activating factor 16
Bahado-Singh *et al.* 2018	serum was mixed with D_2O and buffer	**Non-targeted**<br>NMR (32)<br>Varian Inova 500 MHz	**Serum** samples, stored at -80 °C	60 HW<br>Age: 59.2 ± 12.7,<br>Discovery (training and test set)	46 EC FIGO I-II, 10 EC III-IV<br>Age: 59.1 ± 12.8,	**All EC/HW**<br>Significant differences: 4/32; 36/149 (16 overlap)<br>VIP: 3-hydroxybutyrate, C14:2, C6 (C4:1 DC), C10, C18:2, L-Met, C8, 2-hydroxybutyrate, C7-

Metabolomics [38]<br><br>USA	solution (11.667 mmol disodium-2,2-dimethyl-2-silapentance-5-sulfonate, 730 mmol imidazole, 0.47% NaN_3)	**Targeted** Absolute/IDQ™<br>RP-LC-MS/MS (149) (Biocrates Life Sciences) ABSciex API4000Qtrap		36 HW<br>Validaton:<br>24 HW	Discovery (training and test sets)<br>33<br>Validaton:<br>23 EC	DC, C18:1, C16, kynureine, C14:1, PCae C40:1, acetone<br>**LR model (validation data)**<br>**EC/HW**<br>C14:2, PCae C38:1, 3-hydroxybutyric acid<br>**AUC: 0.83**<br>SEN: 82.6%<br>SP: 70.8%<br>C18:2, PCae C40:1, C6, C4:1-DC<br>**AUC: 0.81**<br>SEN: 82.6%<br>SP: 66.7%<br>BMI, C14:2, PCae C40:1<br>**AUC: 0.80**<br>SEN: 78.3%<br>SP: 62.5%<br><br>**EC stage I-II/ HW**<br>PCae C38:1, 3-hydroxybutyric acid, C14:2<br>**AUC: 0.82**<br>SEN: 72.2%<br>SP: 79.2%<br>BMI, C14:2, PCae C40:1<br>**AUC: 0.80**<br>SEN: 72.2%<br>SP: 75.0%
Shao *et al.* 2016 Clinica Chimica Acta [39]<br><br>China	urine was mixed 100:1 with 100 mmol NaN_3	**Non-targeted** RP-UPLC-ESI-Q-TOF-MS Waters Micromass Q/TOF micro Synapt High Definition MS	**Urine** samples collected in the morning, stored at -80 °C	25 healthy women (HW), 10 patients with endometrial hyperplasia (EH)	25 EC patients<br>no significant difference in age and weight	**PLS-DA model (all 60 patients)**<br>**5 metabolites EC/HW**<br>↓ porphobilinogen, acetylcysteine<br>↑ N-Acetylserine, urocanic acid, isobutyrylglycine<br>**SVM model**<br>**EC/HW+ EH** (2/3 training set, 1/3 test set)
Cheng *et al.* 2019	cervicovaginal fluid was	**Non-targeted** 1H NMR	**Cervicovaginal fluid**	33 Non-EC controls	21 EC patients	Training data set: 17 cases, 28 controls<br>Test data set: 4 cases; 5 controls

Metabolomics [40]<br><br>Taiwan	mixed 2:1 with 0.075 M Na_2HPO_4 pH 7.4 containing 0.08% 3-(trimethylsilyl)-propionic-2,2,3,3,d4 acid sodium salt and 2 mM NaN_3 in D_2O	Bruker Advance 600 MHz	Collected in the middle of the menstrual cycle.	(47 years; range 32-74 years)<br><br>No EC: routine gynaecological check-up; no EC based on final pathology<br><br>Fibroid: 17<br>Endometrioma: 7<br>Adenomyosis: 5<br>Polyp: 4<br><br>No differences in diabetes status, metabolic syndrome, undergoing estroprogestinic therapy	(52 years; range 30-67 years)<br>EC FIGO I: 17<br>EC FIGO II: 1<br>EC FIGO III: 3<br><br>EC grade 1,2: 12<br>EC grade 3: 7	29 metabolites identified<br>Significant ↑: choline, formate, fumarate, malate, phosphocholine<br>Significant ↓: asparagine, aspartate, isoleucine, Phe, pyruvate<br><br>All predicting models built upon phosphocholine, malate, Asp<br>Training:<br>**RF: AUC = 0.92 (0.80-0.99)**<br>**SVM**: AUC = 0.88 (0.76-0.97)<br>**PLS-DA**: **AUC = (0.89 (0.76-0.97)**<br>**LR**: AUC = 0.88 (0.70-0.97)<br>**ANN**: **AUC = 0.88 (0.82-0.92)**<br><br>Testing:<br>**RF**: Acc. 0.78 (0.4-0.97); SEN 0.75 (0.19-0.99); **SP.** 0.8 (0.28-1.00)<br>**SVM**: Acc. 0.78 (0.4-0.97); SEN. 0.75 (0.19-0.99); SP. 0.8 (0.28-1.00)<br>**PLS-DA**: Acc. 0.67 (0.3-0.93); SEN 0.75 (0.19-0.99); SP 0.6 (0.15-0.95)<br>**LR:** Acc. 0.67 (0.3-0.93); SEN 0.75 (0.19-0.999; SP 0.6 (0.15-0.95)<br>**ANN**: Acc. 0.73 (0.63-0.8); SEN. 0.68 (0.55-0.74); SP0.64 (0.52-0.72)	
Trousil *et al.* 2014, Cancer Res. [41]<br><br>UK	tissue was thawed and rinsed with 0.9% NaCl in D_2O	**Non-targeted**<br>1 H-NMR<br>Bruker DRX600	**Endometrial tissue**, frozen in liquid N2 and stored at -80 °C	10 control patients<br>Median age 47.8 years	10 EC patients G3<br>Median age 65.8 years	↑ Val, Leu, Ala, Pro, phosphocholine, Tyr<br>↓ glutathione, scyllo-inositol, myo-inositol, inosine/adenosine	**PLS-DA model**<br>**AUC = 0.987**
Jove *et al.* 2016 Oncotarget [42]	tissue homogenized in 180 mM KCl, 5 mM	**Non-targeted**<br>RP-LC-ESI-QTOF-MS/MS<br>m/z < 3000	**Tissue** samples, fresh-frozen	15 normal endometrium (NE, 10 P, 5 S)	27 EC (endometrioid 6 GI, 13 G II, 8 G III)<br>Two different samples:	**EC/ NE:**<br>53 metabolites<br>↑ stearamide, monoolein, hypoxanthine, 1,2-dihexadecanoyl-sn-glycerol	

Spain	3-[N-morpholino] propanesulfonic acid, 2 mM ethylenediaminetetraacetic acid (EDTA), 1 mM diethylenetriaminepentaacetic acid and 1 mM butylated hydroxyl toluene, 10 mg/ml aprotinin, 1 mM phenylmethylsulfonyl fluoride, pH 7.3; then extracted with methanol	Agilent 6520			Surface endometrioid carcinoma (SEC) and myometrial invasive front (MIF)	**PLS-DA**<br>**G III-IV/ I-II:**<br>27 metabolites<br>↓ Taurine, erythriol,<br>↑ oleamide<br>**SEC/MIF 135 metabolites:**<br>↑ xanthine, lactamide, alpha-D-fucose, 3-mercaptopyruvate, ribitol, PC 32:0, eicosapentaenoic acid<br>↓ inosine, deoxycytidine, hypoxanthine, CDP-ethanolamine, 5-methylthioadenosine
Altadill *et al.* 2017 Scientific Reports [43]<br><br>Spain	tissue was homogenized in 50:50 H_2O:methanol; protein precipitation with acetonitrile; metabolite extraction	**Non-targeted** RP-UPLC-ESI-TOF-MS Waters SYNAPT G2 Si	**Tissue** samples, fresh frozen, stored at -80 °C	17 control women (C), Benign diseases; age > 50, postmenopausal, no treatment	39 EC patients,<br>10 IA, 9 IB, 10 II, 10 III, age >50, postmenopausal, no treatment	**EC/C**<br>80 metabolites, 42 identified mainly lipids ↑ 8 glycerophosphocholines, 1 PS, 1 PG, 9 PE, 4 PI; linoleic acid, 3-deoxyvitamin D3, UDP-N-Acetyl-D-galactosamine, 1-palmitoyl-2-linoleoyl PE<br>↓ Glu-Phe-Arg-Trp, palmic amide, stearamide, oleamide, 1 PAs, 2 PE, PG, inosine, picolinic acid<br>**29 stage I/II EC versus 10 stage III**<br>↑ PC, 2 PEs, ↓ PC, PE, arachidonic acid, UDP-N-acetyl-D- galactosamine

	with dichloromethane:methanol					**Tumor progression: changes in lipidome (PC, PE,** ↑ arachidonic acid
Cummings *et al*. 2019, J. Pathol. [44]<br><br>UK	tissue was homogenized in methanol/water and acidified; solid-phase extraction	**Targeted** RP-LC-MS/MS Waters Quattro Ultima	Endometrial **tissue** frozen	53 normal (NE), 13 P, 6 S, 33 atrophic, 31 atypical hyperplasia<br>Endometrial specimens obtained from women undergoing hysterectomy	108 cancerous tissue, 55 type I (G1, G2); 53 type II (10 G3, 19 serous, 5 clear cell, 4 mixed, 15 carcinosarcoma), 79 FIGO I, 7 II, 14 III, 8 IV, 50 LVSI, 58 no LVSI,<br>Age 67 (39-89)	Dihydro-15-keto derivatives:<br>↓ type I and type II /NE<br>13,14-dihydro-15-keto PGE2<br>↓ type 2 /NE<br>13,14-dihydro-15-keto PGF2α<br>Type II/ type I EC:<br>↓ 12-HETE

Legend: ANN, artificial neural network; DHEA, dehydroepiandrosterone; DHT, dihydrotestosterone; E1, estrone; E2, estradiol; E3, estriol; E1-S, estrone-sulfate; ESI-MS/MS, electrospray ionisation tandem mass spectrometry; G, grade; HRT, hormone replacement therapy; LVSI, lymphovascular space invasion; MeO, methoxy; MD, missing data; MI, myometrial invasion; NA, not available; ND, not determined; OPLS-DA, orthogonal partial least squares discriminant analysis; OC, oral contractption; OR, odds ratio; PCA, Principal Component Analysis; PCae; PCaa, glycerophospholipids; P, proliferative phase; PC, phosphatidylcholine, PE, phosphatidylethanolamine; PG, phosphatidylglycerol; PI, phosphatidylinositol; PLS-DA, Partial Least Squares Discriminant Analysis; RF, random forest; S, secretory phase; SEN, sensitivity; SM, sphingomyelin; SMOH, hydroxysphingomyelin; SP, specificity; SVM; support verctor machine; VIP, variable importance in projection; QTOF, quadrupole time of flight

Supplementary Table S9: PRISMA 2009 Checklist.

Section/topic	#	Checklist item	Reported on page #
**TITLE**			
Title	1	Identify the report as a systematic review, meta-analysis, or both.	1
**ABSTRACT**			
Structured summary	2	Provide a structured summary including, as applicable: background; objectives; data sources; study eligibility criteria, participants, and interventions; study appraisal and synthesis methods; results; limitations; conclusions and implications of key findings; systematic review registration number.	1
**INTRODUCTION**			
Rationale	3	Describe the rationale for the review in the context of what is already known.	1
Objectives	4	Provide an explicit statement of questions being addressed with reference to participants, interventions, comparisons, outcomes, and study design (PICOS).	6
**METHODS**			
Protocol and registration	5	Indicate if a review protocol exists, if and where it can be accessed (e.g., Web address), and, if available, provide registration information including registration number.	6,7
Eligibility criteria	6	Specify study characteristics (e.g., PICOS, length of follow-up) and report characteristics (e.g., years considered, language, publication status) used as criteria for eligibility, giving rationale.	6
Information sources	7	Describe all information sources (e.g., databases with dates of coverage, contact with study authors to identify additional studies) in the search and date last searched.	6
Search	8	Present full electronic search strategy for at least one database, including any limits used, such that it could be repeated.	Table 1
Study selection	9	State the process for selecting studies (i.e., screening, eligibility, included in systematic review, and, if applicable, included in the meta-analysis).	Table 2
Data collection process	10	Describe method of data extraction from reports (e.g., piloted forms, independently, in duplicate) and any processes for obtaining and confirming data from investigators.	7
Data items	11	List and define all variables for which data were sought (e.g., PICOS, funding sources) and any assumptions and simplifications made.	6
Risk of bias in individual studies	12	Describe methods used for assessing risk of bias of individual studies (including specification of whether this was done at the study or outcome level), and how this information is to be used in any data synthesis.	7; Fig. 3, Fig. S1
Summary measures	13	State the principal summary measures (e.g., risk ratio, difference in means).	NA
Synthesis of results	14	Describe the methods of handling data and combining results of studies, if done, including measures of consistency (e.g., I^2) for each meta-analysis.	NA

Supplementary Table S9: PRISMA 2009 Checklist.

Section/topic	#	Checklist item	Reported on page #
Risk of bias across studies	15	Specify any assessment of risk of bias that may affect the cumulative evidence (e.g., publication bias, selective reporting within studies).	6, Figure 3
Additional analyses	16	Describe methods of additional analyses (e.g., sensitivity or subgroup analyses, meta-regression), if done, indicating which were pre-specified.	NA
**RESULTS**			
Study selection	17	Give numbers of studies screened, assessed for eligibility, and included in the review, with reasons for exclusions at each stage, ideally with a flow diagram.	8-9
Study characteristics	18	For each study, present characteristics for which data were extracted (e.g., study size, PICOS, follow-up period) and provide the citations.	9-12
Risk of bias within studies	19	Present data on risk of bias of each study and, if available, any outcome level assessment (see item 12).	12-13
Results of individual studies	20	For all outcomes considered (benefits or harms), present, for each study: (a) simple summary data for each intervention group (b) effect estimates and confidence intervals, ideally with a forest plot.	Tables S5-S8
Synthesis of results	21	Present results of each meta-analysis done, including confidence intervals and measures of consistency.	NA
Risk of bias across studies	22	Present results of any assessment of risk of bias across studies (see Item 15).	Fig. 3, Fig. S1
Additional analysis	23	Give results of additional analyses, if done (e.g., sensitivity or subgroup analyses, meta-regression [see Item 16]).	NA
**DISCUSSION**			
Summary of evidence	24	Summarize the main findings including the strength of evidence for each main outcome; consider their relevance to key groups (e.g., healthcare providers, users, and policy makers).	13-15
Limitations	25	Discuss limitations at study and outcome level (e.g., risk of bias), and at review-level (e.g., incomplete retrieval of identified research, reporting bias).	15-16
Conclusions	26	Provide a general interpretation of the results in the context of other evidence, and implications for future research.	16-17
**FUNDING**			
Funding	27	Describe sources of funding for the systematic review and other support (e.g., supply of data); role of funders for the systematic review.	17

From: Moher D, Liberati A, Tetzlaff J, Altman DG, The PRISMA Group (2009). Preferred Reporting Items for Systematic Reviews and Meta-Analyses: The PRISMA Statement. PLoS Med 6(7): e1000097. doi:10.1371/journal.pmed1000097

For more information, visit: **www.prisma-statement.org**.

References

[1] H.R. Heinonen, M. Mehine, N. Mäkinen, A. Pasanen, E. Pitkänen, A. Karhu, N.S. Sarvilinna, J. Sjöberg, O. Heikinheimo, R. Bützow, L.A. Aaltonen, E. Kaasinen, Global metabolomic profiling of uterine leiomyomas, Br. J. Cancer. 117 (2017) 1855–1864. https://doi.org/10.1038/bjc.2017.361.

[2] K. Vouk, N. Hevir, M. Ribič-Pucelj, G. Haarpaintner, H. Scherb, J. Osredkar, G. Möller, C. Prehn, T.L. Rižner, J. Adamski, Discovery of phosphatidylcholines and sphingomyelins as biomarkers for ovarian endometriosis, Hum. Reprod. 27 (2012) 2955–2965. https://doi.org/10.1093/humrep/des152.

[3] S. Vicente-Muñoz, I. Morcillo, L. Puchades-Carrasco, V. Payá, A. Pellicer, A. Pineda-Lucena, Pathophysiologic processes have an impact on the plasma metabolomic signature of endometriosis patients, Fertil. Steril. 106 (2016) 1733-1741.e1. https://doi.org/10.1016/j.fertnstert.2016.09.014.

[4] S. Letsiou, D.P. Peterse, A. Fassbender, M.M. Hendriks, N.J. van den Broek, R. Berger, O.F. Dorien, A. Vanhie, A. Vodolazkaia, A. Van Langendonckt, J. Donnez, A.C. Harms, R.J. Vreeken, P.G. Groothuis, M.M. Dolmans, A.B. Brenkman, T.M. D'Hooghe, Endometriosis is associated with aberrant metabolite profiles in plasma, Fertil. Steril. 107 (2017) 699-706.e6. https://doi.org/10.1016/j.fertnstert.2016.12.032.

[5] D.P.A.F. Braga, D.A. Montani, A.S. Setti, E.G.L. Turco, D. Oliveira-Silva, E. Borges, Metabolomic profile as a noninvasive adjunct tool for the diagnosis of Grades III and IV endometriosis-related infertility, Mol. Reprod. Dev. 86 (2019) 1044–1052. https://doi.org/10.1002/mrd.23221.

[6] N. Starodubtseva, V. Chagovets, A. Borisova, D. Salimova, N. Aleksandrova, K. Chingin, H. Chen, V. Frankevich, Identification of potential endometriosis biomarkers in peritoneal fluid and blood plasma via shotgun lipidomics, Clin. Mass Spectrom. 13 (2019) 21–26. https://doi.org/10.1016/j.clinms.2019.05.007.

[7] M. Dutta, M. Joshi, S. Srivastava, I. Lodh, B. Chakravarty, K. Chaudhury, A metabonomics approach as a means for identification of potential biomarkers for early diagnosis of endometriosis, Mol. Biosyst. 8 (2012) 3281–3287. https://doi.org/10.1039/c2mb25353d.

[8] S.K. Jana, M. Dutta, M. Joshi, S. Srivastava, B. Chakravarty, K. Chaudhury, 1H NMR based targeted metabolite profiling for understanding the complex relationship connecting oxidative stress with endometriosis, Biomed Res. Int. 2013 (2013). https://doi.org/10.1155/2013/329058.

[9] Y.H. Lee, C.W. Tan, A. Venkatratnam, C.S. Tan, L. Cui, S.F. Loh, L. Griffith, S.R. Tannenbaum, J.K.Y. Chan, Dysregulated sphingolipid metabolism in endometriosis, J. Clin. Endocrinol. Metab. 99 (2014) E1913–E1921. https://doi.org/10.1210/jc.2014-1340.

[10] N. Ghazi, M. Arjmand, Z. Akbari, A.O. Mellati, H. Saheb-Kashaf, Z. Zamani, H NMR- based metabolomics approaches as non-invasive tools for diagnosis of endometriosis, Int. J. Reprod. Biomed. 14 (2016) 1–8.

[11] S. Vicente-Muñoz, I. Morcillo, L. Puchades-Carrasco, V. Payá, A. Pellicer, A. Pineda-Lucena, Nuclear magnetic resonance metabolomic profiling of urine provides a noninvasive alternative to the identification of biomarkers associated with endometriosis, Fertil. Steril. 104 (2015) 1202–1209. https://doi.org/10.1016/j.fertnstert.2015.07.1149.

[12] K. Vouk, M. Ribič-Pucelj, J. Adamski, T.L. Rižner, Altered levels of acylcarnitines,

phosphatidylcholines, and sphingomyelins in peritoneal fluid from ovarian endometriosis patients, J. Steroid Biochem. Mol. Biol. 159 (2016) 60–69. https://doi.org/10.1016/j.jsbmb.2016.02.023.

[13] F. Domínguez, M. Ferrando, P. Díaz-Gimeno, F. Quintana, G. Fernández, I. Castells, C. Simón, Lipidomic profiling of endometrial fluid in women with ovarian endometriosis, Biol. Reprod. 96 (2017) 772–779. https://doi.org/10.1093/biolre/iox014.

[14] V. V. Chagovets, Z. Wang, A.S. Kononikhin, N.L. Starodubtseva, A. Borisova, D. Salimova, I.A. Popov, A. V. Kozachenko, K. Chingin, H. Chen, V.E. Frankevich, L. V. Adamyan, G.T. Sukhikh, Endometriosis foci differentiation by rapid lipid profiling using tissue spray ionization and high resolution mass spectrometry, Sci. Rep. 7 (2017) 1–10. https://doi.org/10.1038/s41598-017-02708-x.

[15] M. Dutta, B. Singh, M. Joshi, D. Das, E. Subramani, M. Maan, S.K. Jana, U. Sharma, S. Das, S. Dasgupta, C.D. Ray, B. Chakravarty, K. Chaudhury, Metabolomics reveals perturbations in endometrium and serum of minimal and mild endometriosis, Sci. Rep. 8 (2018) 1–9. https://doi.org/10.1038/s41598-018-23954-7.

[16] J. Li, Y. Gao, L. Guan, H. Zhang, J. Sun, X. Gong, D. Li, P. Chen, Z. Ma, X. Liang, M. Huang, H. Bi, Discovery of phosphatidic acid, phosphatidylcholine, and phosphatidylserine as biomarkers for early diagnosis of endometriosis, Front. Physiol. 9 (2018) 1–7. https://doi.org/10.3389/fphys.2018.00014.

[17] J. Li, L. Guan, H. Zhang, Y. Gao, J. Sun, X. Gong, D. Li, P. Chen, X. Liang, M. Huang, H. Bi, Endometrium metabolomic profiling reveals potential biomarkers for diagnosis of endometriosis at minimal-mild stages, Reprod. Biol. Endocrinol. 16 (2018) 1–10. https://doi.org/10.1186/s12958-018-0360-z.

[18] C.L. Feider, S. Woody, S. Ledet, J. Zhang, K. Sebastian, M.T. Breen, L.S. Eberlin, Molecular Imaging of Endometriosis Tissues using Desorption Electrospray Ionization Mass Spectrometry, Sci. Rep. 9 (2019) 1–11. https://doi.org/10.1038/s41598-019-51853-y.

[19] A. Hasim, M. Ali, B. Mamtimin, J.Q. Ma, Q.Z. Li, A. Abudula, Metabonomic signature analysis of cervical carcinoma and precancerous lesions in women by 1H NMR spectroscopy, Exp. Ther. Med. 3 (2012) 945–951. https://doi.org/10.3892/etm.2012.509.

[20] A. Hasim, A. Aili, A. Maimaiti, B. Mamtimin, A. Abudula, H. Upur, Plasma-free amino acid profiling of cervical cancer and cervical intraepithelial neoplasia patients and its application for early detection, Mol. Biol. Rep. 40 (2013) 5853–5859. https://doi.org/10.1007/s11033-013-2691-3.

[21] Y. Hou, M. Yin, F. Sun, T. Zhang, X. Zhou, H. Li, J. Zheng, X. Chen, C. Li, X. Ning, G. Lou, K. Li, A metabolomics approach for predicting the response to neoadjuvant chemotherapy in cervical cancer patients, Mol. Biosyst. 10 (2014) 2126–2133. https://doi.org/10.1039/c4mb00054d.

[22] M. zhu Yin, S. Tan, X. Li, Y. Hou, G. Cao, K. Li, J. Kou, G. Lou, Identification of phosphatidylcholine and lysophosphatidylcholine as novel biomarkers for cervical cancers in a prospective cohort study, Tumor Biol. 37 (2016) 5485–5492. https://doi.org/10.1007/s13277-015-4164-x.

[23] K. Yang, B. Xia, W. Wang, J. Cheng, M. Yin, H. Xie, J. Li, L. Ma, C. Yang, A. Li, X. Fan, H.S. Dhillon, Y. Hou, G. Lou, K. Li, A Comprehensive Analysis of Metabolomics and Transcriptomics in Cervical Cancer, Sci. Rep. 7 (2017) 1–11. https://doi.org/10.1038/srep43353.

[24] I. Khan, M. Nam, M. Kwon, S.S. Seo, S. Jung, J.S. Han, G.S. Hwang, M.K. Kim, Lc/ms-based

polar metabolite profiling identified unique biomarker signatures for cervical cancer and cervical intraepithelial neoplasia using global and targeted metabolomics, Cancers (Basel). 11 (2019). https://doi.org/10.3390/cancers11040511.

[25] H. Zhou, Q. Li, T. Wang, H. Liang, Y. Wang, Y. Duan, M. Song, Y. Wang, H. Jin, Prognostic biomarkers of cervical squamous cell carcinoma identified via plasma metabolomics, Medicine (Baltimore). 98 (2019) e16192. https://doi.org/10.1097/MD.0000000000016192.

[26] N. Ye, C. Liu, P. Shi, Metabolomics analysis of cervical cancer, cervical intraepithelial neoplasia and chronic cervicitis by 1H NMR spectroscopy, Eur. J. Gynaecol. Oncol. 36 (2015) 174–180. https://doi.org/10.12892/ejgo2613.2015.

[27] H.M. Woo, K.M. Kim, M.H. Choi, B.H. Jung, J. Lee, G. Kong, S.J. Nam, S. Kim, S.W. Bai, B.C. Chung, Mass spectrometry based metabolomic approaches in urinary biomarker study of women's cancers, Clin. Chim. Acta. 400 (2009) 63–69. https://doi.org/10.1016/j.cca.2008.10.014.

[28] Z.E. Ilhan, P. Łaniewski, N. Thomas, D.J. Roe, D.M. Chase, M.M. Herbst-Kralovetz, Deciphering the complex interplay between microbiota, HPV, inflammation and cancer through cervicovaginal metabolic profiling, EBioMedicine. 44 (2019) 675–690. https://doi.org/10.1016/j.ebiom.2019.04.028.

[29] A.O. Tokareva, V. V. Chagovets, N.L. Starodubtseva, N.M. Nazarova, M.E. Nekrasova, A.S. Kononikhin, V.E. Frankevich, E.N. Nikolaev, G.T. Sukhikh, Feature selection for OPLS discriminant analysis of cancer tissue lipidomics data, J. Mass Spectrom. 55 (2020). https://doi.org/10.1002/jms.4457.

[30] A. Abudula, N. Rouzi, L. Xu, Y. Yang, A. Hasimu, Tissue-based metabolomics reveals potential biomarkers for cervical carcinoma and HPV infection, Bosn. J. Basic Med. Sci. 20 (2020) 78–87. https://doi.org/10.17305/bjbms.2019.4359.

[31] Y. Ihata, E. Miyagi, R. Numazaki, T. Muramatsu, A. Imaizumi, H. Yamamoto, M. Yamakado, N. Okamoto, F. Hirahara, Amino acid profile index for early detection of endometrial cancer: Verification as a novel diagnostic marker, Int. J. Clin. Oncol. 19 (2014) 364–372. https://doi.org/10.1007/s10147-013-0565-2.

[32] T. Knific, K. Vouk, Š. Smrkolj, C. Prehn, J. Adamski, T.L. Rižner, Models including plasma levels of sphingomyelins and phosphatidylcholines as diagnostic and prognostic biomarkers of endometrial cancer, J. Steroid Biochem. Mol. Biol. 178 (2018) 312–321. https://doi.org/10.1016/j.jsbmb.2018.01.012.

[33] E. Strand, I.L. Tangen, K.E. Fasmer, H. Jacob, M.K. Halle, E.A. Hoivik, B. Delvoux, J. Trovik, I.S. Haldorsen, A. Romano, C. Krakstad, Blood metabolites associate with prognosis in endometrial cancer, Metabolites. 9 (2019). https://doi.org/10.3390/metabo9120302.

[34] Y. Audet-Delage, J. Grégoire, P. Caron, V. Turcotte, M. Plante, P. Ayotte, D. Simonyan, L. Villeneuve, C. Guillemette, Estradiol metabolites as biomarkers of endometrial cancer prognosis after surgery, J. Steroid Biochem. Mol. Biol. 178 (2018) 45–54. https://doi.org/10.1016/j.jsbmb.2017.10.021.

[35] Y. Audet-Delage, L. Villeneuve, J. Grégoire, M. Plante, C. Guillemette, Identification of metabolomic biomarkers for endometrial cancer and its recurrence after surgery in postmenopausal women, Front. Endocrinol. (Lausanne). 9 (2018) 1–12. https://doi.org/10.3389/fendo.2018.00087.

[36] J. Troisi, L. Sarno, A. Landolfi, G. Scala, P. Martinelli, R. Venturella, A. Di Cello, F. Zullo, M.

Guida, Metabolomic Signature of Endometrial Cancer, J. Proteome Res. 17 (2018) 804–812. https://doi.org/10.1021/acs.jproteome.7b00503.

[37] K. Shi, Q. Wang, Y. Su, X. Xuan, Y. Liu, W. Chen, Y. Qian, G.E. Lash, Identification and functional analyses of differentially expressed metabolites in early stage endometrial carcinoma, Cancer Sci. 109 (2018) 1032–1043. https://doi.org/10.1111/cas.13532.

[38] R.O. Bahado-Singh, A. Lugade, J. Field, Z. Al-Wahab, B.S. Han, R. Mandal, T.C. Bjorndahl, O. Turkoglu, S.F. Graham, D. Wishart, K. Odunsi, Metabolomic prediction of endometrial cancer, Metabolomics. 14 (2018) 1–9. https://doi.org/10.1007/s11306-017-1290-z.

[39] X. Shao, K. Wang, X. Liu, C. Gu, P. Zhang, J. Xie, W. Liu, L. Sun, T. Chen, Y. Li, Screening and verifying endometrial carcinoma diagnostic biomarkers based on a urine metabolomic profiling study using UPLC-Q-TOF/MS, Clin. Chim. Acta. 463 (2016) 200–206. https://doi.org/10.1016/j.cca.2016.10.027.

[40] S.C. Cheng, K. Chen, C.Y. Chiu, K.Y. Lu, H.Y. Lu, M.H. Chiang, C.K. Tsai, C.J. Lo, M.L. Cheng, T.C. Chang, G. Lin, Metabolomic biomarkers in cervicovaginal fluid for detecting endometrial cancer through nuclear magnetic resonance spectroscopy, Metabolomics. 15 (2019). https://doi.org/10.1007/s11306-019-1609-z.

[41] S. Trousil, P. Lee, D.J. Pinato, J.K. Ellis, R. Dina, E.O. Aboagye, H.C. Keun, R. Sharma, Alterations of choline phospholipid metabolism in endometrial cancer are caused by choline kinase alpha overexpression and a hyperactivated deacylation pathway, Cancer Res. 74 (2014) 6867–6877. https://doi.org/10.1158/0008-5472.CAN-13-2409.

[42] M. Jové, S. Gatius, A. Yeramian, M. Portero-Otin, N. Eritja, M. Santacana, E. Colas, M. Ruiz, R. Pamplona, X. Matias-Guiu, Metabotyping human endometrioid endometrial adenocarcinoma reveals an implication of endocannabinoid metabolism, Oncotarget. 7 (2016) 52364–52374. https://doi.org/10.18632/oncotarget.10564.

[43] T. Altadill, T.M. Dowdy, K. Gill, A. Reques, S.S. Menon, C.P. Moiola, C. Lopez-Gil, E. Coll, X. Matias-Guiu, S. Cabrera, A. Garcia, J. Reventos, S.W. Byers, A. Gil-Moreno, A.K. Cheema, E. Colas, Metabolomic and Lipidomic Profiling Identifies the Role of the RNA Editing Pathway in Endometrial Carcinogenesis, Sci. Rep. 7 (2017) 1–13. https://doi.org/10.1038/s41598-017-09169-2.

[44] M. Cummings, K.A. Massey, G. Mappa, N. Wilkinson, R. Hutson, S. Munot, S. Saidi, D. Nugent, T. Broadhead, A.I. Wright, S. Barber, A. Nicolaou, N.M. Orsi, Integrated eicosanoid lipidomics and gene expression reveal decreased prostaglandin catabolism and increased 5-lipoxygenase expression in aggressive subtypes of endometrial cancer, J. Pathol. 247 (2019) 21–34. https://doi.org/10.1002/path.5160.
